# Applications of Graphene Quantum Dots in Biomedical Sensors

**DOI:** 10.3390/s20041072

**Published:** 2020-02-16

**Authors:** Bhargav D. Mansuriya, Zeynep Altintas

**Affiliations:** Technical University of Berlin, Straße des 17. Juni 124, 10623 Berlin, Germany; b.mansuriya@campus.tu-berlin.de

**Keywords:** graphene quantum dots (GQDs), nanomaterials, biosensors, optical sensors, electrochemical sensors, photoelectrochemical sensors, biomedical applications

## Abstract

Due to the proliferative cancer rates, cardiovascular diseases, neurodegenerative disorders, autoimmune diseases and a plethora of infections across the globe, it is essential to introduce strategies that can rapidly and specifically detect the ultralow concentrations of relevant biomarkers, pathogens, toxins and pharmaceuticals in biological matrices. Considering these pathophysiologies, various research works have become necessary to fabricate biosensors for their early diagnosis and treatment, using nanomaterials like quantum dots (QDs). These nanomaterials effectively ameliorate the sensor performance with respect to their reproducibility, selectivity as well as sensitivity. In particular, graphene quantum dots (GQDs), which are ideally graphene fragments of nanometer size, constitute discrete features such as acting as attractive fluorophores and excellent electro-catalysts owing to their photo-stability, water-solubility, biocompatibility, non-toxicity and lucrativeness that make them favorable candidates for a wide range of novel biomedical applications. Herein, we reviewed about 300 biomedical studies reported over the last five years which entail the state of art as well as some pioneering ideas with respect to the prominent role of GQDs, especially in the development of optical, electrochemical and photoelectrochemical biosensors. Additionally, we outline the ideal properties of GQDs, their eclectic methods of synthesis, and the general principle behind several biosensing techniques.

## 1. Introduction

Cancer and cardiovascular disorders are becoming the leading causes of death all over the world. In 2018, a global statistical estimation of around 9.6 million human deaths and 18.1 million cancer cases was reported [1], whereas cardiovascular disorders result in nearly 66% of deaths that account for considerable rates of mortality and morbidity [2,3]. Contemporaneously, various neurodegenerative diseases, autoimmune disorders and infectious diseases are threatening modern health care system. Considering the severity of such pathophysiologies as well as their dramatic rise, researchers have zeroed in on the breakthrough inventions of several portable as well as versatile sensing systems for the speedy, highly selective and quite specific determination of the target biomolecules in environmental and clinical areas. Early sensing of these analytes is a key step toward abolishing metastasis, adapting dynamic therapies and preventing fatality. Despite the availability of several conventional culturing methods for sensing target analytes, their limitations reside in tedious and time-consuming procedures, expensive instrumentation, highly skilled manpower, low sensitivity and impotency to perform in-field monitoring. Henceforth, there is a huge demand to achieve an on-site detection of target biomolecules in biological media and thereby to contrive the necessary precautions for their effective inactivation. This has now become remarkably convenient by virtue of biosensor development.

### 1.1. History, Definition and Classification of Biosensors

In 1962, Clark and Lyons invented the very first biosensor that was an amperometric enzyme-based sensor for monitoring glucose levels [4]. Since then, biosensors have garnered tremendous attention for biomedical applications, especially for drug discovery, disease monitoring and quantification of target analytes. These analytes generally include microorganisms (i.e., viruses, bacteria, fungi, etc.), biomarkers responsible for causing certain diseases, environmental toxins, pollutants, allergens and metal ions. The reminiscent biosensors are reported to determine these target analytes in biological matrices including human blood, sweat, saliva, urine, food products, environmental samples, etc. [5,6,7,8,9,10].

Biosensors are analytical tools comprising a biological recognition element and a suitable transducer that are usually connected to an appropriate data-processing system [11]. These sensors integrate a biological element with a physiochemical transducer to generate an electronic signal, which is directly proportional to the analyte concentration and subsequently conveyed to a detector [12]. Generally, the classification of biosensors depends on: (a) the type of receptors employed during the bio-recognition events, such as antibody [13], peptide [14], enzyme [15,16] aptamer [17], DNA [18] and molecularly imprinted polymer (MIP)-based sensors [19,20]; (b) the type of transducers involved, such as electrochemical [21], optical [22], piezoelectric [23] and calorimetric biosensors [24]. They can accomplish the requirements of rapid and specific sensing of the biomolecules as well as the real-time analysis of modern detection techniques. Thus, it is speculated that these sensing platforms can provide abundant opportunities for research and development.

### 1.2. Role of Nanomaterials in Biosensing

Nanomaterials are nano-sized (1–100 nm) materials with three-dimensional (3D) space [25]. In the current scenario, the research regarding various nanomaterials is evolving immensely in such a way that they are successfully becoming an element in our routine lifestyle in terms of food safety, environmental sciences, cosmetics, therapeutics, drug delivery, biosensors, etc. [26,27,28]. With these, routes for the divulgence of nanomaterials to humans and ecological systems are increasing. Such nanomaterials have been extensively explored in modifying electrode surfaces to fabricate biosensors for their further improvement with respect to the critical features like reproducibility, selectivity and sensitivity, owing to their excellent biocompatibility, structural compatibility and strong adsorption capability [29]. They exhibit unique biological and physicochemical characteristics, macroscopic quantum tunneling effects, surface effects and small size effects. Therefore, they are quite distinct from their conventional counter-parts as far as their optical, electrical, magnetic, mechanical and catalytic behaviors are concerned. This makes them suitable for the development of biosensors [30,31,32,33]. Additionally, they are often used for signal amplification by serving as nanocarriers including electron transfer promoters, nanozymes, detector bioreceptors, electroactive labeling elements, and catalysts [34,35,36,37], hence offering novel strategies for biosensing platforms and their practical applicability. Over the last decade, numerous nanomaterials have been continuously studied and employed as signal-amplifying species such as nanoparticles (NPs) [38,39,40], graphene [41,42,43], nanowires [44], carbon nanotubes (CNTs) [45], magnetic beads [46,47] and quantum dots (QDs) [48,49]. Among these nanomaterials, QDs such as graphene quantum dots (GQDs) and carbon dots (CDs) are becoming quite well-known for their multifarious properties such as signal amplifying characteristics, good biocompatibility, tunable size, electro-catalytic performance as well as their capacity for the concurrent and multiple detection of biomolecules. Moreover, their robustness, inertness, non-toxicity, long-term chemical stability, water-solubility as well as their photo-stability against both photo-bleaching and blinking are some of the typical characteristics that are considered for their applications in biomedicine. Nevertheless, they can be readily functionalized and their synthetic procedures are quite effortless [49,50,51,52,53,54,55].

### 1.3. Ideal Properties of Graphene Quantum Dots (GQDs)

Being zero-dimensional (0D), GQDs are carbon-based anisotropic nanomaterials constituting a fabric-like structure homologous to graphene. The morphological features of GQDs imitate both CDs as well as graphene [56]. They are broadly employed as smart probes for environmental, optoelectronics, electrochemical and biological operations [49,57,58,59]. Their edge dimension is bigger than their vertex, rendering single or manifold panels of graphene with chemical moieties on their lateral surface, which deliver a large number of sites for chemical functionalization [49]. They can be easily conjugated with several nanomaterials via π–π interaction, with a purpose to generate hybrid nanomaterials [60]. Furthermore, GQDs can also be grafted with antibodies, proteins and small-nucleic acids due to their dimensional resemblance to such molecules. They can effectively enhance the surface of biosensors for absorbing a noticeable number of receptors [54,61,62].

Innumerable nanomaterials exhibiting stable and strong fluorescence, including carbon nanomaterials, up-conversion nanoparticles, metal nanoparticles, polymer-encapsulated organic nanoparticles, inorganic silica nanoparticles and semiconductor QDs have been reported for their efficient biosensing applications. Among these, semiconductor QDs were so far preferred as ideal fluorescent probes owing to their narrow range of emission, broad excitation wavelength, good photo-stability and easy functionalization ability with surface reagents. Nonetheless, most of the semiconductor QDs (i.e., PbS, CdTeSe@CdZnS, CdTe@CdSe and CdHgTe) comprise heavy metals, resulting into unfavorable environmental and biological detrimental effects, which obstruct their biomedical applicability [63,64,65]. However, GQDs being a member of the carbon family have emerged as novel carbon-based fluorescent materials, due to their excellent biocompatibility, fluorescence property and non-toxicity. These features of GQDs make them ideal nanomaterials for biomedical applications over other semiconductor nanomaterials.

According to several studies, when the light-emitting attributes of GQDs have been taken into consideration, their origin is still unclear because their extrinsic states seem to be derived from undesired impurities and foreign moieties, although their luminescent property primarily originates from quantum confinement. In close proximity, the influence of oxygen functionalities on the luminescence of GQDs must be thoroughly investigated to understand its luminescent behavior [66].

Exclusively, GQDs can act as nanozymes or electro-catalysts for catalyzing hydrogen peroxide (H_2_O_2_) to determine target analytes via a label-free approach [67]. They exhibit peroxidase (POD)-like catalytic features that result in a redox reaction between H_2_O_2_ and an electron-donating substrate. In the sector of biosensors, an enzyme called horseradish peroxidase (HRP) is often associated with the labeling of secondary bioreceptors for the corresponding analyte determination, which in turn makes the assays more tedious and costlier [18]. To carry out these procedures faster and cost-effectively, GQDs are now utilized for replacing HRP-conjugated secondary bioreceptors [53,67,68].

GQDs serve as attractive fluorophores, i.e., as fluorescent labels, quenchers and energy as well as charge donors [69]. They are fluorescent nanosized graphene segments, which lead to quantum-size effects and exciton confinement in the range from 3 to 20 nm particles [70,71]. This is considering the fact that graphene exhibits zero band, produces non-luminescence and possess a ‘Bohr radius’ with an infinite excitation that exhibit quantum confinement in defined sized particles [72]. Contradictorily, GQDs possess a band-gap due to their quantum confinement, lateral effects and size-effects that can be readily altered by virtue of their size and edge chemistry [57,72]. In contrast to semiconductor QDs, which acquire double quantum states (qs.) at a certain level of energy, GQDs have twice more (i.e., 4 qs.). These surplus qs. make GQDs suitable for computational quantum analysis [49,60].

### 1.4. Approaches to Synthesize GQDs for Biomedical Sensors

GQDs can be prepared using a broad range of methods, some of which are elaborated in the ensuing sections along with their role in the respective biosensor development. Nonetheless, GQDs with distinguishable and adjustable sizes can be chemically prepared by following one of two strategies:

(a) In a top-down approach, graphene oxide (GO) or 2D graphene sheets, CNTs, graphite or carbon fibers are fragmented to obtain 0D GQDs [73,74,75].
(b) In a bottom-up approach, synthesis of GQDs is usually conducted by a series of chemical reactions engaging small molecular precursors [58,71].

**Figure 1:**
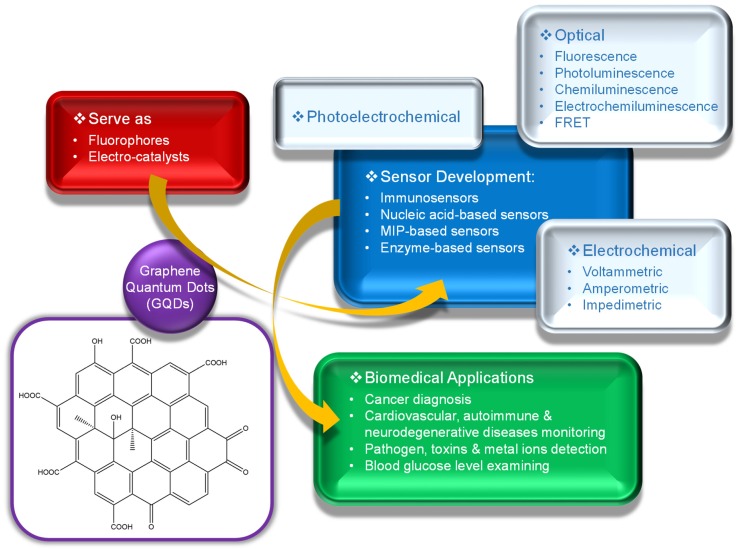
The chemical structure of graphene quantum dots (GQDs) and their biomedical applications in sensor development.

Since 2015, many investigational studies on GQD biosensors have made a giant step forward due to their aforementioned characteristics [67,76,77,78]. These studies could specifically and sensitively detect target analytes by virtue of multiplexing electrodes such as screen-printed carbon electrodes (SPCE), screen-printed gold electrodes (SPGE) glassy carbon electrodes (GCE), etc. These sensing platforms have been validated to perform cost-effective, trustworthy, utterly sensitive and exact detection of target biomolecules for governing healthcare systems [79]. Moreover, some biosensors have been reported to offer multiplexed ability for concurrent detection of multiple biomolecules [80,81]. To comprehend the recent achievements, main features and general working principle behind the GQD biosensors reported from 2015 onwards, we have discussed them in the ensuing sections; according to the transducer type, i.e., optical, electrochemical and photoelectrochemical, and sensors for biomedical applications. The reviewed GQD-sensing techniques and their biomedical applications are outlined in Figure 1.

## 2. Optical GQD Sensors in Biomedical Diagnostics

Optical biosensors are among the well-established sensors for determining target biomolecules. Commonly, they bring together an emitting light source, a modulating element, a bio-receptor and a photo-detector for interpreting the optical response of a generated signal [11,82]. Optical transducers include fluorescence, photoluminescence (PL), chemiluminescence (CL), electrochemiluminescence (ECL), fluorescence resonance energy transfer (FRET) or Förster resonance energy transfer, interferometry, optical wavelength-modulated spectroscopy and surface plasmon resonance (SPR) [11,82,83,84,85,86,87]. These techniques interpret optical signals that are highly sensitive to the refractive index variation in the proximity of bio-recognition events [86].

### 2.1. Fluorescence-Based GQD Sensors

Fluorescence is a process with a short life-span of luminescence generated due to the creation of electromagnetic excitation upon a material absorbing higher light energy and consecutively, emitting lower light energy, i.e., at a shorter and longer wavelengths, respectively [88,89,90]. In fluorescence, duration from the absorption till emission phenomena is within just a fraction of a second, ranging from 10^−9^ to 10^−8^ s. [91]. When GQDs are employed for biomedical applications, besides excitation wavelength, ambient pH should also be considered, since it determines the quantum efficiency of the fluorescence excitation [92]. It is worth mentioning that there have been plenty of GQD sensors reported in last few years displaying the role of their fluorescent characteristics.

In 2019, Li et al. synthesized pentaethylenehexamine and histidine-functionalized graphene quantum dots (PEHA-GQD-His) [93]. The as-prepared PEHA-GQD-His served as the fluorescence probe for a microRNA (miRNA) fluorescence biosensing nanoplatform assembled with a molecular beacon double-cycle amplification approach. The study involved the specific binding of the target microRNA to the molecular beacon to provoke the target cycle and the molecular beacon cycle, generating a DNA nanoassembly on the PEHA-GQD-His film. The terminated G-quadruplexes could fold and fuse to hemin well enough to establish hemin/G-quadruplex complexes. Thereafter, the fluorescence emission intensity of PEHA-GQD-His could be quenched through photoinduced electron transfer by an electron acceptor and hemin anchored on the PEHA-GQD-His, which resulted in an in situ development by the H_2_O_2_ decomposition, because of the G-quadruplex/hemin DNAzymes’ catalytic performance. This approach indicates that the design of a target and beacon double cycle can promote specific DNA nanoassembly on the PEHA-GQD-His surface, whereas the DNAzyme-attenuated double-quenching mechanism can attain fluorescence-quenching ability of PEHA-GQD-His. Moreover, the presence of PEHA elevates the fluorescence emission intensity of PEHA-GQD-His, where histidine increases the catalysis of G-quadruplex/hemin DNAzymes towards H_2_O_2_. This biosensor could deliver a sensitive fluorescence response towards miRNA in human serum in a linear calibration range of 1 × 10^−18^–1 × 10^−12^ M and could achieve 4.3 × 10^−19^ M as the limit of detection (LOD).

In 2016, Laurenti et al. reported a biosensor using single stranded DNA (ssDNA) and GQDs, which were coupled with the up-conversion nanoparticles (UCNPs) and silica (SiO_2_) for determining miRNA sequences [94]. The sensor involved the interaction of sp^2^ carbon atoms of the GQDs with ssDNA‒UCNPs through π–π stacking, which could bring GQDs to the ssDNA‒UCNP/SiO_2_ surface. This led to an increase in the up-conversion emission and at the same time, hybridization of the ssDNA with their correspondent complementary miRNA sequences hindered UCNPs to react with the GQDs via π–π interaction. This resulted in a diminished fluorescence emission intensity depending on the concentration of miRNA sequences. This strategy for developing a sensing platform for miRNA sequences could achieve an LOD of 10 fM.

**Figure 2:**
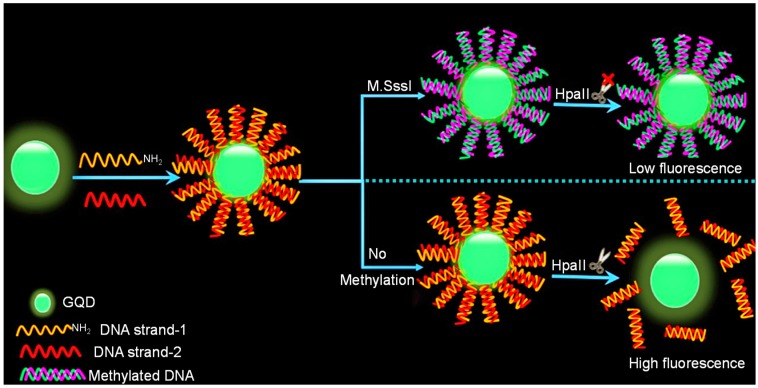
Schematic representation of a fluorescent assay for DNA methyltransferase activity [95].

Since DNA methyltransferase (M.SssI) enzyme plays a significant role in several biological processes and its abnormal expression can cause cancer, Kermani and the team introduced a biosensing method for monitoring the activity of this enzyme in human serum [95]. This method was based on DNA-functionalized GQDs for a fluorescence-based assay as shown in Figure 2. An aminated double stranded-DNA (ds-DNA) was formed that constituted a recognition site for M.SssI as well as endonuclease HpaII. Upon the fusion of ds-DNA to GQDs, a significant reduction in fluorescence intensity of about 45% was observed, while ds-DNA was methylated upon the conjugation with M.SssI, leading to develop resistant to cleavage by HpaII and no change in fluorescence. On the other hand, in the absence of M.SssI enzyme, HpaII could easily cleave ds-DNA and resulted in an increased fluorescence. This method could detect M.SssI with an LOD of 0.7 U mL^−1^. Moreover, fluorescence anisotropy was also conducted for confirming the DNA modification and liberation of ds-DNA from the GQDs’ surface in the existence of M.SssI and HpaII, respectively.

A label-free GQD-based ratiometric fluorescence assay was proposed for detecting the DNA by merging the cutting endonuclease enzyme supported with the hemin DNAzyme/G-quadruplex bio-catalysis via the target recycling as well as a cascade signal amplification strategy [96]. In the study, o-phenylenediamine (OPD) was chosen as a hemin DNAzyme/G-quadruplex substrate, where 2,3-diaminophenazine (DAP), i.e., the oxidized product, could quench the fluorescence of GQDs (~460 nm), coupled with a fresh emission of DAP (~564 nm). Such a ratiometric signal (i.e., I_564_/I_460_) could sensitively and specifically determine target DNA, with an LOD of 30 fM.

In 2017, Sun and co-workers constructed a biosensor for determining cholesterol using hydrothermally synthesized nitrogen-doped GQDs (N‒GQDs) having quantum yield (QY) of 80%. These N‒GQDs were coupled with chromium picolinate (CrPic), where the researchers explored the fluorescence-quenching effect of the CrPic/N‒GQDs complex. [97]. The research involved the implantation of CrPic on to the N‒GQDs through a cross-linking agent, cysteamine (Cys) as well as the quenching of the N‒GQDs’ fluorescence intensity. This was achieved via a phenomenon called ‘photoinduced electron transfer’ (PET), where CrPic and N‒GQDs acted as the electron-donating and electron-accepting groups, respectively. Subsequently, cholesterol was introduced to form a CrPic/N‒GQDs complex, since CrPic could serve as an efficient cholesterol-receptor as well, through affinity binding as well as π–π stacking. A significant enhancement in fluorescence emission intensity of CrPic/N‒GQDs implied the ability of cholesterol to enhance the conductivity of N‒GQDs. The authors stated that this CrPic/N‒GQDs-based biosensor could clinically detect cholesterol in human serum, where the linear concentration range and LOD were observed to be 0–520 mM and 0.4 mM, respectively.

Using europium (Eu)-macromolecule complex, Ryu et al. functionalized GQDs with two different sizes that were employed for developing a fluorescent sensor for *Bacillus anthracis* spores [98]. This GQDs‒Eu dual emission biosensor possessed high sensitivity, morphology of ultrafine particles, improved dispersibility and enhanced surface-to-volume ratio. The GQDs‒Eu displayed multiple emission bands, which could be attributed to the fluorescence emitted from the red dipicolinic acid–Eu (DPA–Eu) complex (i.e., ~593 nm and ~616 nm) as well as from the blue GQDs (~435 nm). As a consequence, GQDs were introduced as a non-interfering internal calibration to form a ratiometric sensor. The time-dependent fluorescence relationship confirmed the completion of a reaction, which facilitated the rapid detection of *B. anthracis* spores within 8 s. The as-prepared Eu‒GQDs sensor could demonstrate the quantification of *B. anthracis* with an LOD of around 10 pM, which was 6-fold less than the infectious dose of *B. anthracis* spores. Moreover, the cross-reactivity study revealed that GQDs‒Eu sensors could show selectivity of about 103 times for DPA in comparison to the competing aromatic ligands.

Ascorbic acid (AA) is a vitamin, which has a pivotal function in several physiological reactions occurring in living cells and abnormal levels can cause various diseases. Thus, it is crucial to develop potent methods than can accurately determine the AA level in human cells. In 2017, Feng and the team found that the near-infrared (NIR)‒GQDs could produce a two-photon (TP) excitation with cross-section (δΦ) of 25.12 Goeppert–Mayer units and generated an NIR peak (~660 nm) upon excitation with 810 nm fs pulses [99]. Having exhibited TP fluorescence characteristics, these NIR‒GQDs were used to fabricate a TP nanoprobe for detecting endogenous AA in the human body. Herein, NIR‒GQDs that act as fluorescence reporters, possessed lower fluorescence background that could sharpen the fluorescence-imaging resolution. Moreover, cobalt oxyhydroxide (CoOOH) nanoflakes served as fluorescence quenchers by fusing with the NIR‒GQDs surface. In the presence of AA, CoOOH was converted to Co^2+^ via a reduction reaction, which generated a “turn-on” fluorescence signal of NIR‒GQDs. This nanosystem could susceptibly detect AA with an LOD of 270 nM.

Another biosensor based on the fluorescence turn-on assay approach for determining AA concentrations in human serum was also reported in 2017 [100]. In this study, the researchers investigated the orange emission of GQDs and the role of HRP as well as H_2_O_2_. Injection of HRP and H_2_O_2_ oxidized catechol resulted in the conversion of o-benzoquinone that could effectively quench the fluorescence of GQDs. Nonetheless, when AA was introduced, H_2_O_2_ and hydroxyl radicals were consumed that led to the inhibition of o-benzoquinone production, resulting in fluorescence recovery. Fluorescence emission intensity gave a linear correlation for H_2_O_2_ concentrations from 3.33 to 500 µM, while that with the AA concentrations from 1.11 to 300 µM. The detection limits for H_2_O_2_ and AA were observed to be 1.2 µM and 0.32 µM, respectively.

In 2018, Na et al. established a detection strategy for AA as well as alkaline phosphatase (ALP) enzyme, where a fluorescence “turn off-on” assay was designed through the in situ formation of MnO_2_ nanosheets with sulfanilic acid functionalized GQDs (Sa‒GQDs) [101]. In the study, ALP could catalyze the hydrolysis of amifostine to S-2-(3-aminopropylamino)-ethanethiol (molecule A), and the addition of KMnO_4_-produced MnO_2_ nanosheets and molecule B (i.e., the polyamine disulfide form of molecule A). Subsequently, the energy transfer platform was constructed by adhering Sa‒GQDs to MnO_2_ nanosheets through molecule B as a crosslinking reagent, which resulted in the fluorescence quenching of Sa‒GQDs by MnO_2_ nanosheets. AA led to the decomposition of MnO_2_ into Mn^2+^ owing to its extraordinary reducing ability and disintegrated the MnO_2_ nanosheets that could liberate Sa‒GQDs, thereby recovering the quenched fluorescence. Additionally, both the ALP as well as AA generated the change in color of solution due to the redox reaction of MnO_2_ nanosheets. Therefore, MnO_2_ nanosheets could also be employed as colorimetric probes for the quantification of ALP and AA via direct visualization through the naked eye. This Sa‒GQDs/KMnO_4_/amifostine/ALP system provided a linearity, ranging from 0.5 to 20 μmol L^−1^ AA concentration, with the quantification limit of 0.16 μmol L^−1^.

On the basis of a chemical redox strategy for modulating the fluorescence of N‒GQDs, a biosensor for tracing the ALP concentration has been proposed [102]. Initially, the fluorescence of N‒GQDs was effectively quenched by ultrathin CoOOH nanosheets, followed by the restoration through AA that could convert CoOOH to Co^2+^ by reduction reaction. Hence, the ALP activity was sensed, depending on the hydrolytic performance of ALP for converting L-ascorbic acid-2-phosphate (AAP) to AA by ALP. This label-free biosensor could quantify the ALP concentration, ranging from 0.1 to 5 U L^−1^ with an LOD of 0.07 U L^−1^. Another label-free biosensing platform for ALP activity determination was reported, where GQDs with high QY were synthesized by a single-step reaction [103]. In the study, two linear calibration plots were observed, ranging from 0.05 to 2.5 nM and 5 to 10 nM, respectively. The optimized values implicated that this nanoprobe could detect ALP with an LOD of 0.017 nM.

**Figure 3:**
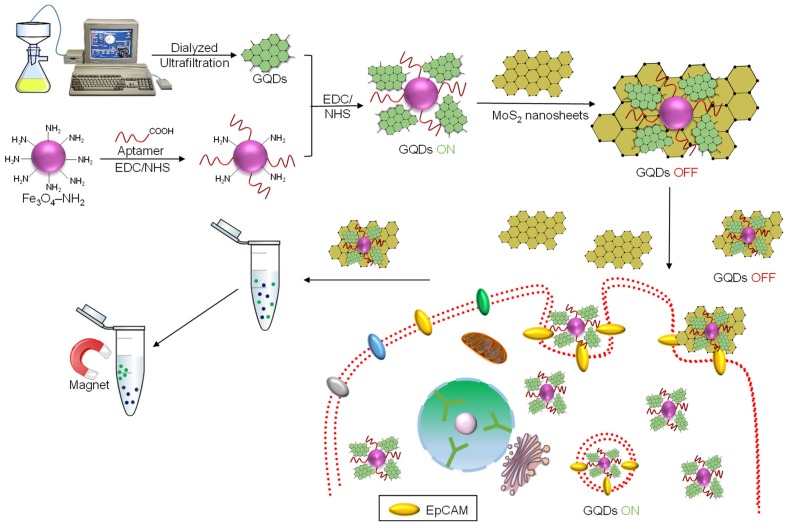
The detection principle of the aptamer/Fe_3_O_4_/GQDs/MoS_2_-based nanosurface energy transfer biosensor for sensing circulating tumor cells (CTCs).

In 2019, Cui et al. introduced a concept of a ‘turn-on’ magnetic fluorescent biosensor (Figure 3) using molybdenum disulfide (MoS_2_) nanosheets, Fe_3_O_4_ and GQDs for detecting circulating tumor cells (CTCs) [104]. In this approach, electrochemically synthesized GQDs were modified with a magnetic agent and EpCAM (epithelial cell adhesion molecule) aptamer. MoS_2_ nanosheets act as a fluorescence quencher, which was coupled with the GQD/Fe_3_O_4_/EpCAM aptamer complex. This fusion led to the formation of ‘turn-on’ biosensing magnetic fluorescent nanocomposites (MFNs). These MFNs render a negligible cellular toxicity with around 90% of an average capture efficacy (i.e., higher than that of other magnetic NPs). Moreover, the MFNs-based biosensor could rapidly sense and label CTCs within 15 min, beating several other steps of detection techniques. In the presence of EpCAM aptamers, the MFNs are specific for capturing CTCs (i.e., both low- and high-EpCAM-expressing cells). It was reported that this strategy could detect up to 10 tumor cells in human blood with a linear detection range and detection limit of 2–64 nM and 1.19 nM, respectively.

Dopamine (DA), the catecholamine neurotransmitter has a pivotal function in regulating hormones as well as in metabolizing cells. Moreover, it acts as a biomarker in diseases related to its secretion, which include Parkinson’s disease, Huntington’s disease, schizophrenia, senile dementia, anorexia etc. [105,106] Therefore, it is essential to monitor DA levels for which numerous GQD sensors based on fluorescence have already been reported.

Zhou and co-workers constructed an MIP-based sensor for mapping DA using polyindole (PIn) and GQDs [107]. The PIn/GQDs@MIPs sensing system could readily bind DA and showed a high sensitivity for DA concentrations with a wide linear array from 5 × 10^−10^ to 1.2 × 10^−6^ M and an LOD of 1 × 10^−10^ M, owing to the tailor-made imprinted cavities via hydrogen bonds between O_2_-rich groups of the nanocomposite and amine groups of DA. Additionally, this sensor could rebind DA in dual-type: a high-affinity type and a low-affinity type (i.e., when non-covalent interaction is “on” and “off”, respectively), where the rebinding step could be controlled through pH modulation, suggesting distinct binding efficiency for tuning the binding interaction. This research group had also demonstrated a fluorescent sensing strategy for DA determination with the use of polypyrrole (PPy) and GQDs core/shell hybrids [108]. These nanocomposites delivered a strong fluorescence emission, which was 3 orders of magnitudes higher than that by pristine GQDs. This sensor could result in a fluorescent emission intensity falling off with the increasing DA concentrations in the range of 5 to 8000 nM. Moreover, the quantification limit for DA was down to 10 pM. Later on, in 2017, the same group used MIP/GQDs and poly(indolylboronic acid) (PIn-BAc) for the identification of DA [105]. While preparing the MIPs@PIn-BAc/GQDs system, the introduction of DA led to the fluorescence quenching of the nanocomposites as well as agglomeration due to the covalent interaction between boronic acid and catechol group of DA. The results revealed that this system could also provide a wide linear range of DA concentration, ranging from 5.0 nM to 1.2 μM and the detection limit of 2.5 nM. Moreover, it was reported that all these three sensors could perform well against several interfering biomolecules. Hence, they can exhibit high specificity and can be employed for the clinical testing of DA in human biological media.

In 2016, Zhao et al. fabricated a label-free technique, taking GQDs as effective probes for the quantification of DA [109]. GQDs gave a strong blue fluorescence in aqueous solution, which was then quenched by adding DA. The quenching mechanism was based on the transfer of electrons from the photo-excited GQDs to DA–quinine that was generated through DA oxidization by O_2_ in alkaline environment. The quenched fluorescence was directly co-related with the DA concentration in the range of 0.25 to 50 µM, with an LOD of 0.09 µM. In the same year, Tashkhourian and Dehbozorgi synthesized GQDs via control carbonization of citric acid for testing DA levels in human serum [110]. Herein, DA could quench the fluorescence emission intensity of GQDs through dynamic quenching. This technique attained a calibration plot for DA in a linear array of 0.01–50.0 µM, achieving the low quantification limit of 8.2 nM and exhibited high specificity for DA in the co-existence of interfering compounds like uric and ascorbic acid.

Xiaoyan et al. studied the role of GO‒GQDs and N,S‒GQDs in performing HRP modification to determine H_2_O_2_ in real water samples [111]. These GQDs were produced by dissecting GO as well as by pyrolyzing citric acid and L-cysteine, which revealed an antagonistic influence on the HRP activity. However, the GO‒GQDs-functionalized HRP exhibited an excellent thermo-stability and improved activity (i.e., 1.9 order of magnitudes higher than pristine enzyme), which can be attributed to the bigger conjugate rigid plane and lesser hydrophilic groups delivered by GO‒GQDs in contrast to the N,S‒GQDs. Such features of GO‒GQDs can make them suitable for offering a desired conformational change in HRP for catalysis, enhanced thermo-stability as well as enzymatic performance. The HRP functionalized by GO‒GQDs was also used for bio-catalysis to probe H_2_O_2_ by a fluorescence biosensor. It was observed that transparent tetramethylbenzidine (TMB) was oxidized into blue-colored TMB in the co-existence of H_2_O_2_ by support of HRP functionalized with GO‒GQDs, resulting in the quenching of GO‒GQDs’ fluorescence. The fluorescence emission intensity was decreased in a linear fashion with increasing concentration of H_2_O_2_, ranging from 2 nM to 200 μM and an LOD value of 0.68 nM was achieved.

The nanoprobe development for TP microscopy is in huge demand to screen various biomolecules in humans, but these nanoprobes are limited to single-color fluorescence variations, which make them inappropriate for quantitative analysis. To overcome these limitations, a rational dual-emission and TP-GQDs probe for sensing H_2_O_2_ has been reported [112]. Herein, a boronate ester-modified merocyanine (BMC) fluorophore was employed as target-activator and as a dual-emission fluorescence modulator to specifically recognize H_2_O_2_ and to quench the fluorescence of TP-GQD. Upon TP excitation (~740 nm), TP-GQD−BMC revealed a green-to-blue colored fluorescence corresponding to H_2_O_2_ with a shift in emission (~110 nm), and the H_2_O_2_ could be successfully probed in the range of 0.2–40 μM, with a detection limit of 0.05 μM.

In 2018, Qu et al. designed a biosensor for mapping phytic acid (PA) and H_2_O_2_ exploiting the role of glutathione (GSH)-modified GQDs [113]. The fluorescence of GQDs@GSH was quenched by Fe^3+^ ions through a PET process. When the PA was introduced to GQDs@GSH/Fe^3+^ system, the fluorescence of GQDs@GSH was considerably recovered by the assistance of the strong reducing and chelating efficiency of PA, where Fe^3+^ ions were reduced to Fe^2+^ ions by PA and led to the development of a PA/Fe^2+^ complex. Such an ‘off–on’ fluorescence strategy was proposed for determining PA owing to the use of GQDs@GSH/Fe^3+^ as a fluorescent probe. Moreover, the same strategy could also detect H_2_O_2_, where H_2_O_2_ could inactivate the chelate structure of PA/Fe^2+^, liberate Fe^2+^ ions and oxidize Fe^2+^ ions to generate Fe^3+^ ions, resulting in the fluorescence quenching of GQDs@GSH. This approach could serve a broad linear response for both PA and H_2_O_2_ in the range of 0.05 to 3 µmol L^−1^ and 0.5 to 10 µmol L^−1^, respectively. The estimated LOD values of PA and H_2_O_2_ were reported to be 14 nmol L^−1^ and 0.134 µmol L^−1^, respectively.

**Figure 4:**
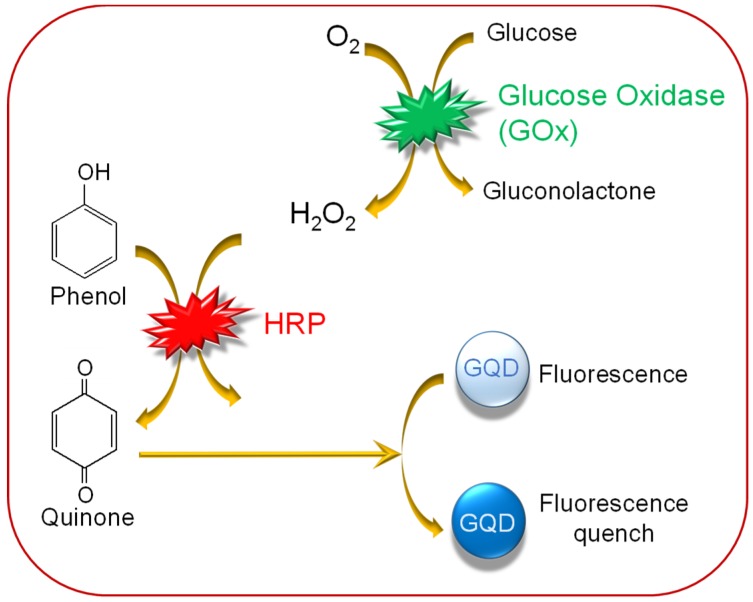
Detection mechanism of an enzyme based glucose-sensor. Adapted from Wang et al. [114].

A GQD-biosensor based on the synergetic effect of enzyme-coupled technique and fluorescence quenching was engineered for monitoring glucose levels in human serum samples [114]. As explained in Figure 4, oxidation of glucose was achieved by the glucose oxidase (GOx) enzyme, producing H_2_O_2_. Subsequently, H_2_O_2_ could oxidize phenol to quinone in the presence of HRP for potential quenching of the GQDs–GOx–HRP–phenol system. Such a sensing system could effectively determine blood glucose levels with a linear relationship between the glucose concentration and the fluorescence intensity, ranging from 0.2–10 µmol L^−1^ and an achievement of 0.08 µmol L^−1^ as the detection limit. Another glucose sensor employing the GOx enzyme and GQDs was developed, where the researchers used hemin-functionalized GQDs [115]. The GQDs were synthesized through the pyrolysis of citric acid, which were highly water-soluble and highly fluorescent. Because of non-covalent binding between hemin and GQDs, hemin could help H_2_O_2_ to demolish the GQD-surface that quenched the fluorescence emission intensity of GQDs. The results displayed a wide linear range of glucose from 9 to 300 µM, with an LOD of 0.1 µM.

In the last few years, GQD-sensors based on fluorescence have been successfully proved to quantify variety of metal ions such as Ag^+^, Cu^2+^, Pb^2+^, Hg^2+^ and Fe^3+^ ions. These sensors could be applied for environmental safety to detect metal ions in water samples as well as for clinical diagnosis of several cancer-types. For example, tracing the Fe^3+^ ion levels in human serum can help in cancer diagnosis. In the development of such sensors, either GQD itself or its functionalized versions with GO (graphene oxide), nitrogen, sulfur, boron, rhodamine, dopamine, amino acids like valine or even when coupled with nanomaterials, e.g., silver nanoparticles (AgNPs), gold nanoparticles (AuNPs), sodium citrate-functionalized up-conversion nanoparticles (Cit-UCNPs), etc. have resulted in the enhanced features of the respective biosensors, which are briefly illustrated in Table 1.

Apart from the aforementioned GQD sensors, numerous other sensors based on fluorescence have also been reported recently for a wide range of biomedical applications. For instance, efficient cancer diagnosis can be achieved through the development of GQD-biosensors by detecting biothiols (e.g., GSH, cysteine or homocysteine) in human serum [131]; an “ON-OFF” biosensor employing GQDs and gold nanocrosses (AuNCs) for tracking intracellular adenosine triphosphate (ATP) concentrations [132]; N–GQDs with a vanadium pentoxide (V_2_O_5_) nanosheet-implanted sensing platform for cysteine [133]; a N–GQD/silica based MIP-sensor for cytochrome-C (Cyt-C) [134]; a selenium-implanted GQD-sensor for the simultaneous determination of oxidative hydroxyl radical (^•^OH) and reductive GSH in HeLa cells [135]; as well as for mapping various enzymes such as trypsin and tyrosinase using a Cyt-C induced GQD-sensor [136]; and an N–GQDs decorated biosensor [137], respectively. A sensor coupled with tyramine-functionalized GQDs has been reported to screen metabolites (i.e., glucose, cholesterol, L-lactate, xanthine) for monitoring several metabolic disorders like diabetes, obesity, lactic acidosis, gout and hypertension [138]. Also, pharmaceutical agents like tacrine (a cholinesterase inhibitor for treating patients with neurodegenerative disorders) and triclosan (an antibacterial drug) can be quantified by virtue of biosensors involving the role of N–GQDs/acetylcholinesterase (achE) enzyme [139] and silicon–GQDs assembled with ruthenium (III) ions [140], respectively. Fluorescence-based GQD-sensors could also quantify pathogens like tuberculosis causing CFP-10 (culture filtrate protein) [141] and water pollutants like trinitrophenol (TNP) [142]. Furthermore, the critical features of some GQD-sensors based on the employed receptor-type are listed in Table 2.

### 2.2. Photoluminescence-Based GQD-Sensors

Photoluminescence (PL) is also a phenomenon involving light emission from fluorophores or fluorochromes like GQDs after the absorption of photons (i.e., electromagnetic radiation). It is one of the types of luminescence, which is promoted by photoexcitation. In other words, it is a process of photons that excite electrons to a higher energy level in an atom [89,90]. The working principle behind PL sensors resembles that of fluorescence-based sensors. Although PL and fluorescence are similar to each other, we categorize these sensors separately according to the terminologies reported in the respective research works.

**Figure 5:**
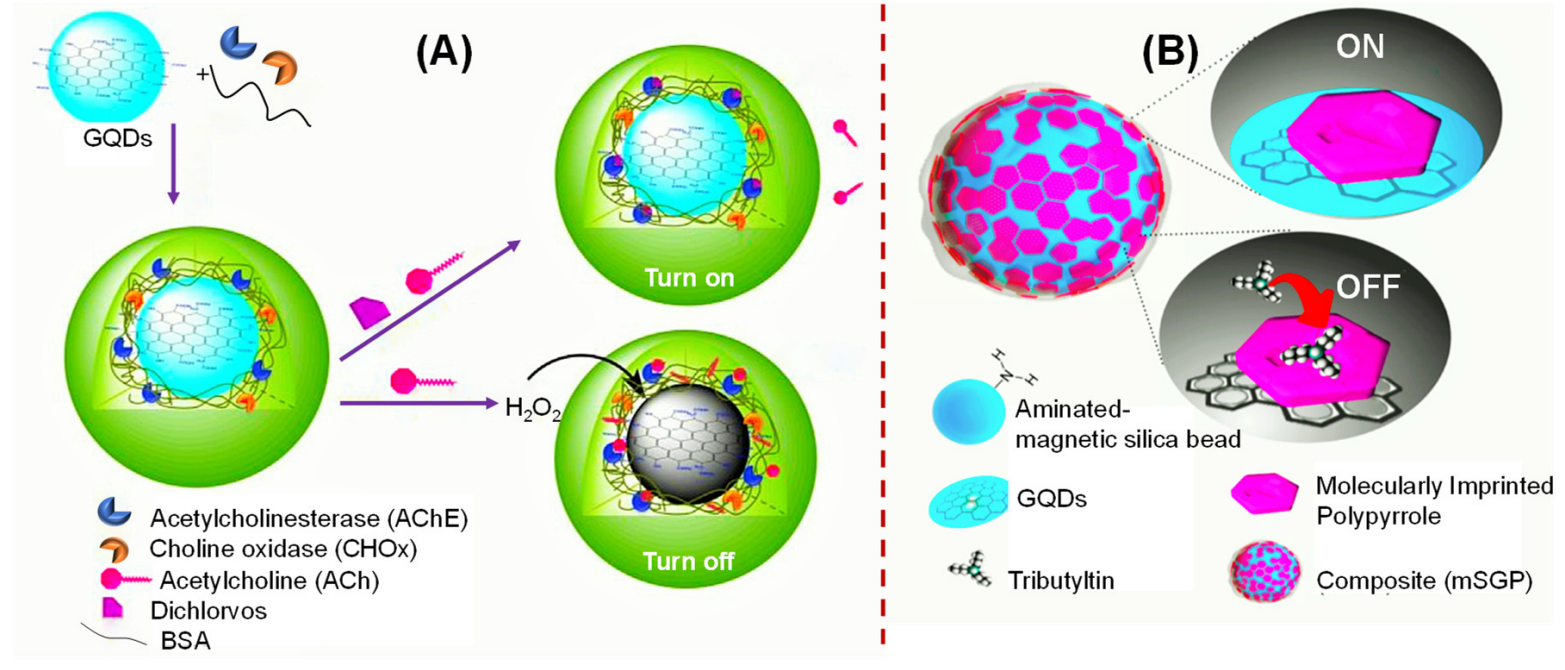
Schematic representation of: (**A**) Enzyme-based photoluminescence (PL) sensor for dichlorvos [156]; (**B**) MIP-based PL sensor for tributyltin [157].

In 2018, Sahub et al. designed an enzyme-based sensor for the detection of a pesticide, dichlorvos (i.e., an organophosphate) as represented in Figure 5A [156]. This strategy involved the production of H_2_O_2_ from the enzymatic reaction of acetylcholinesterase (AChE) and choline oxidase (CHOx), which could react with GQDs to generate a “turn-off” photoluminescence of GQDs and could be restored at 467 nm in the presence of dichlorvos. The change in PL intensity of the GQDs/AChE/CHOx system was directly proportional to the concentration of dichlorvos and could detect a lower amount of dichlorvos down to 0.172 ppm (~0.778 μM). Such a facile and cost-effective biosensor was developed to interrogate the presence of organophosphate pesticides in food, water and the environment. Another GQD-sensor for the determination of pesticides was constructed by Zor et al. as shown in Figure 5B [157]. Herein, a multifunctional nanocomposite, i.e., magnetic silica beads/GQDs/molecularly imprinted polypyrrole (mSGP), was synthesized for specifically capturing and signaling small molecules owing to the synergism offered by this composite in terms of optical, chemical and magnetic features along with molecular imprinting of tributyltin, a genotoxic substance that can cause endocrine disruptions. Moreover, its magnetic property could be employed for capturing and pre-concentrating tributyltin on the surface, and the PL intensity of GQDs was effectively quenched during the binding of tributyltin. This sensing technique could quantify tributyltin in water and seawater without pretreating the samples and achieved the LOD values of 12.78 and 42.56 ppb, respectively.

Zhao et al. investigated a signaling transduction approach based on PL to screen immunoglobulin G (IgG) via the interaction between graphene (Gr) and GQDs [158]. To engineer the immunosensor, mouse IgG (mIgG) antibody–GQDs and Gr were selected as donors and acceptors, respectively. On introducing Gr to the mIgG–GQDs solution, the non-specific binding interaction between the Gr surface and mIgG as well as the π–π stacking between GQDs and Gr could easily bring Gr and GQDs together in luminescence resonance energy transfer (LRET) to promote the luminescence quenching of GQDs depending on LRET. The detection step involved the specific binding of human IgG to mIgG with the assistance of an immunoreaction that led to the Gr surface being far away from mIgG–GQDs, which restricted the LRET and recovered the luminescence of GQDs. The increase in PL intensity was directly correlated with the human IgG concentration, ranging from 0.2 to 12 μg mL^−1^, with an estimated LOD of 10 ng mL^−1^.

In 2018, by using N,S‒GQDs, Mondal and team provided a biosensing strategy to measure the concentrations of nitroexplosive, 2,4,6-trinitrophenol (TNP) [159]. These GQDs could generate abundant localized energy levels near the conduction band. Only a 90 µM solution of TNP could result in a considerable improvement in fluorescence quenching of N,S‒GQDs (~92%), as compared to their individual doped states (i.e., either by using N‒GQDs or S‒GQDs). This could be attributed to the charge transfer among these doped states to selectively determine TNP. The detection limit was reported to be 19.05 ppb. He et al. demonstrated a facile PL method for the identification of hydroquinone (H_2_Q), exploiting the use of GQDs that act as peroxidase-mimicking catalyst as well as PL indicator [160]. When the dissolved oxygen was present, GQDs could oxidize H_2_Q to p-benzoquinone that could quench PL of GQDs. This nanosensing PL platform for H_2_Q provided an LOD of 5 nM.

A facile mix-and-detect PL technique was introduced for the turn-on mapping of acidic amino acids like glutamic acid (Glu) and aspartic acid (Asp) [161]. Prior to the detection strategy, GQDs that emit both up-conversion and down-conversion PL intensities were synthesized using the solvothermal method. The carboxylic acid-rich surface enhanced the water solubility of the GQDs as well as being able to trigger Eu^3+^‒GQDs aggregation, which led to the effective PL quenching of GQDs. The quenched PL was restored by competing acidic amino acids with GQDs for Eu^3+^. The sensitive and specific analysis of acidic amino acids was reported on the basis of both the up- and down-conversion PL and it was clearly proved from this research that up-conversion mode delivers a little lower LOD than the down-conversion process. The detection of Glu was accomplished by up-conversion PL of GQDs, where the PL intensity steeply increased with the Glu concentrations from 1 to 200 μM, with an LOD of 0.19 μM. On the other hand, the down-conversion mode for Glu could also follow the same linear detection range, but with an LOD of 0.32 M. This can be assigned to the greater interference from the background in down-conversion mode. Under the similar conditions, nearly identical results were observed for Asp, i.e., a linear detection range from 1 to 220 μM, with LOD values of 0.18 μM and 0.33 μM for up-conversion and down-conversion modes, respectively.

**Figure 6:**
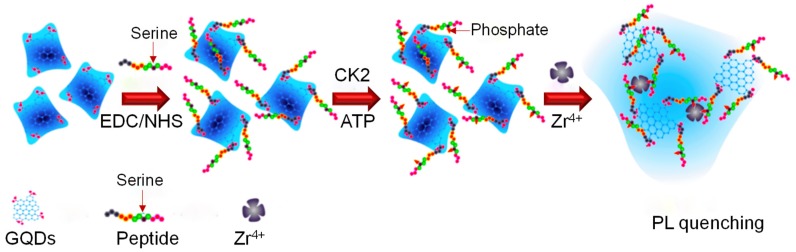
A GQD sensor based on PL for screening protein kinase activity [162].

Figure 6 embodies a PL assay for monitoring the protein kinase activity depending on the selective agglomeration of a phosphorylated peptide−GQD complex triggered by Zr^4+^ ions [162]. This bioassay showed a decrease in the PL intensity of peptide−GQD complex for casein kinase II (CK2) concentrations, ranging from 0.1 to 1.0 unit mL^−1^, with an LOD of 0.03 unit mL^−1^. Moreover, the EC_50_ value (i.e., defined as the concentration at which 50% of an enzyme can be converted to substrate) for CK2 was calculated as 0.34 unit mL^−1^. The as-developed assay can be applied for the effective screening of kinase inhibitor within 5 min. To validate the ability of this GQD-based sensor for monitoring the kinase inhibition activity in complex biological matrices like human serum, the inhibitory activity of CK2 phosphorylation was demonstrated by various inhibitors such as ellagic acid, emodin, quercetin and 5,6-dichlorobenzimidazole-l-β-d-ribofuranoside. The results obtained suggested that the PL intensity increases with increasing inhibitor efficiency in the presence of the aforementioned inhibitors. For example, the IC_50_ (i.e., inhibitor concentration resulting in 50% inhibition) in case of ellagic acid was found as 0.041 × 10^−6^ M, implying that this assay can be a promising approach to quantify the enzymes as well as their inhibitors with satisfactory results.

Ju et al. used hydrothermally prepared N‒GQDs having QY of 32.4% (at 350 nm) by following a one-step reaction of dicyandiamide and citric acid for determining GSH levels in HeLa cells. [163]. The quenching of the PL intensity of bright luminescent N‒GQDs could be successfully achieved by Hg^2+^ because of the electron transfer as well as the strong electrostatic interaction between N‒GQDs and Hg^2+^ ions, which was then restored by adding GSH due to the selected combination of GSH and Hg^2+^ ions via an Hg^2+^‒S bond. This turn-on fluorescence sensor provided magnificent selectivity and sensitivity for GSH, and an LOD of 87 × 10^−9^ M. Safardoust-Hojaghan et al. also carried out hydrothermal synthesis of GQDs with 25% QY, but using citric acid and ethylenediamine for quantifying bacterial pathogens such as *Escherichia coli* (*E. coli*) and *Staphylococcus aureus* (*S. aureus*). The authors confirmed the linear relationship between fluorescence intensity of GQDs and concentrations of both *S. aureus* as well as *E. coli* up to 9 × 10^7^ cfu mL^−1^ [164].

Zhang et al. prepared boron-doped GQDs (B‒GQDs) from boron-doped graphene [165]. The presence of boronic acid groups on the surface of B‒GQDs could generate PL for label-free glucose detection. The study revealed that the reaction between two boronic acid groups on B‒GQDs surfaces and two cis-diol units of glucose facilitate conformationally rigid B‒GQDs/glucose agglomerates. This constrains the intramolecular rotation, thereafter leading to an enhanced PL intensity. Moreover, the results proved that B‒GQDs could be highly specific only to glucose rather than its isomeric cousins like fructose, mannose and galactose due to their ability to avoid saccharides with only one cis-diol unit. The PL intensity of this sensor was boosted with elevated glucose concentrations, suggesting a good sensing performance with a linear range from 0. 05 to 10 mM and an LOD of 0.03 mM. The same group had developed a GQD-sensor for determining not only glucose but also H_2_O_2_ [166]. Herein, the production of multifunctional and non-covalent hybrids was achieved through π–π stacking and electrostatic interactions between GQDs and Fe^3+^ 5,10,15,20-tetrakis(1-methyl-4-pyridyl)porphine (FeTMPyP). The PL of GQDs was quenched by the inner filter effect (IFE) of FeTMPyP on the GQDs. The quenched PL of GQDs can then be switched back “on” according to the reaction occurring between FeTMPyP and H_2_O_2_ that created rupture of the cyclic tetrapyrrolic nucleus. Subsequently, Fe^3+^ ions from FeTMPyP were lost and generated colorless mono- and dipyrroles. This “turn-on” sensing system could offer a linear calibration plot for glucose and H_2_O_2_ concentrations from 3 to 100 µM and 2 to 300 µM, respectively, with corresponding LOD values of 0.5 and 0.3 µM. Shehab et al. designed a non-enzymatic glucose sensor with the use of phenylboronic acid receptor-modified GQDs, where the PL of GQDs was considered as the crucial optical parameter for glucose [167]. This sensor could exhibit a good linear relationship for glucose concentrations, ranging from 4 to 40 mM (~72 to 720 mg dL^−1^), with an LOD value of 3.0 mM.

**Figure 7:**
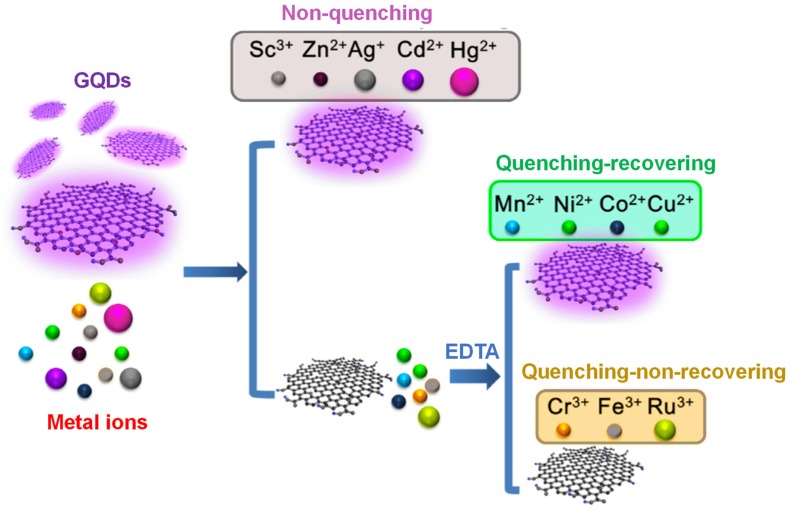
Schematic illustration of various transition metal ions and their influences on the PL of GQDs [168].

As shown in Figure 7, Huang and the group introduced a novel PL-based electron-transfer method, depending upon the quenching of various transition metal ions on the PL intensity of GQDs [168]. In this work, ethylene diamine tetra-acetic acid (EDTA) could competitively interact with metal ions for restoring the quenched PL of GQDs. It was experienced that those metal ions with empty or fully filled d-orbits could not quench the PL emission intensity of GQDs, but those with partly filled d-orbits could do so. With these facts, an optical metal sensing system was designed by considering Ni^2+^ as a model analyte and by employing Ni^2+^-specific chelating reagent (i.e., dimethyl-glyoxime) to substitute EDTA, which could estimate the Ni^2+^ concentration with an LOD of 4.1 μM.

Ananthanarayanan et al. exemplified a GQD-based PL strategy for sensing Fe^3+^ ions [169]. In this research, 0D GQDs were chemically prepared from 3D graphene by the vapor deposition method, where the sensor was capable of detecting Fe^3+^ ions, ranging from 0 to 80 μM and an LOD of 7.22 μM. Another PL-sensor for Fe^3+^ ions was reported with even lower LOD value (i.e., 5 nM). [170]. In this work, GQDs were synthesized by carbonizing the precursors of polycyclic aromatic hydrocarbon (PAH) precursors with strong acid and, consecutively, by reducing hydrothermally with the use of hydrazine hydrate. In 2016, an environment-friendly approach using coffee beans and a hydrazine hydrate-associated hydrothermal method for synthesizing GQDs was reported for Fe^3+^ and Cu^2+^ ions [171]. These GQDs were then modified with polyethylene imine that displayed improved band-edge PL with single exponential decay. Moreover, this sensing platform could be able to detect Fe^3+^ as well as Cu^2+^ ions with an LOD of 1 μM for both. Sun et al. fabricated a PL based GQD-sensor that could quantify the lowest concentrations of Cu^2+^ ions with the detection limit of ca. 7 nM, which could be due to the involvement of amino-modified GQDs [172].

Bai and co-workers established a PL sensor for detecting phosphate ions using GQDs [173]. The off–on sensing principle depended on the combination of GQDs and Eu^3+^ ions, where phosphate ions could be detected by competing O_2_-donor moieties from phosphate ions with those from the ester (RCOOR’) groups on the GQD-surface for Eu^3+^ ions. This sensor could display a broad linear response between the increased PL intensity of GQDs and phosphate ions in the range of 0.5–190 µM, with an LOD of 0.1 µM.

In 2017, Patra and colleagues presented the preparation of GQDs combined with graphene, where the GQDs were hydrothermally sourced from carrot juice [174]. In order to achieve the maximum PL intensity from graphene/GQDs, the graphene sheets were chemically modified with cadmium sulphide (CdS). This nanohybrid material was then functionalized to induce multi-functionalities, for an instance, initiation of polymerization and imprinting of nimesulide polymer. This MIP-sensor could detect nimesulide, with an LOD down to 6.65 ng L^−1^ and can be clinically applied for tracking nimesulide in complex biological matrices including human blood serum, urine samples and pharmaceutical tablets.

### 2.3. Chemiluminescence-Based GQD-Sensors

Chemiluminescence (CL) is the emission of light (luminescence) and/or heat via a chemical reaction. CL can assure high sensitivity (10^−6^–10^−15^ g), and lower detection limits (10^−18^ g) [175]. CL can be deprived of selectivity when it is used directly as a spectrometric method. The instrumentation of CL sensors is pretty simple. Although CL-based biosensors may provide good sensitivity, their selectivity is not good enough. It is therefore essential to improve the selectivity of the CL reaction in the future. The merits of CL sensors are the broad dynamic ranges, quite low detection limits, as well as the comparatively facile instrumentation needed. CL is used as a mechanism that allows quantification of several biomolecules at minimum concentrations. Demerits of such biosensors include insufficient sensitivity and selectivity due to several physical and chemical parameters [176]. Hence, to the best of our knowledge, very few CL based GQD sensors have been developed so far.

**Figure 8:**
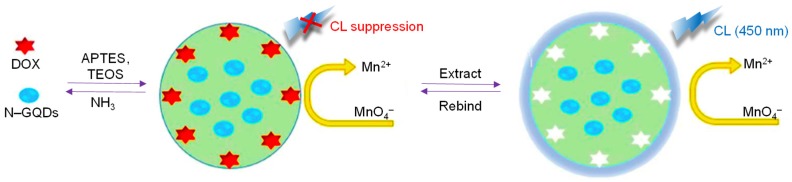
Construction of MIP@N‒GQDs based chemiluminescence (CL) sensor for the quantification of doxorubicin [178]. Reproduced by permission of The Royal Society of Chemistry. APTES: Aminopropyltriethoxysilane; TEOS: Tetraethoxysilane.

Amjadi et al. presented a GQDs-Ce (IV) CL system to quantify uric acid in urine and plasma samples, where the CL of GQDs was generated by direct chemical oxidation [177]. In the study, GQDs were synthesized by a simple carbonization method and characterized by X-ray diffraction (XRD), Fourier transform infrared spectroscopy (FT-IR), transmission electron microscopy (TEM) and Raman spectroscopy. The fluorescence and CL emission spectra were tracked to study the CL generation mechanism, since Ce (IV) can oxidize GQDs to induce a relatively intense CL emission as well as considering the combined property of the radiative oxidant-injected holes and thermally excited electrons in GQDs. This analytical model could determine uric acid, based on its diminishing effect on the GQDs-Ce (IV) CL system, which showed a wide linear response from 1.0 × 10^−6^ M to 5.0 × 10^−4^ M with an LOD of 5.0 × 10^−7^ M. Later, the same group synthesized MIP@N‒GQDs using a facile sol–gel phenomenon for sensing doxorubicin, an anti-cancer drug [178]. Potassium permanganate (KMnO_4_) was added to the MIP@N‒GQDs solution, which emitted a strong CL and on injecting doxorubicin, the CL intensity was considerably quenched to establish a CL detection system for this drug (Figure 8). The mechanism behind the MIP@NGQDs/KMnO_4_ CL system was examined by studying fluorescence, CL as well as ultraviolet–visible (UV–Vis) spectra. The relative CL intensity suppressed in a linear fashion with the doxorubicin concentration over a wide range of 20–260 mg L^−1^ and an LOD value of 4.7 mg L^−1^ was obtained. This strategy was applied for determining therapeutic levels of doxorubicin in a spiked human serum sample.

In 2017, Chen and co-workers demonstrated a Cu^2+^ catalyzed persistent CL sensor for ascorbic acid (AA) determination [179]. This system was constructed using N‒GQDs as a green luminophor, which could emit CL with the direct oxidation by H_2_O_2_. Additionally, the Cu^2+^ ion offers distinct specificity against other interfering metal ions and inflates over twice the CL intensity of N‒GQDs, owing to its catalytic Fenton-like reaction for H_2_O_2_ decomposition. As a result, the scintillating luminescence of N‒GQDs was augmented and then varied to retain perpetuity with minute decay in the existence of the Cu^2+^ ion, possessing the ability for CL visual imaging. This sensor based on Cu^2+^/N‒GQDs/H_2_O_2_ provided quantitative analysis of AA in fruit, displaying a dynamic range and an LOD of 1–100 μM and 0.5 μM, respectively.

In 2018, Hassanzadeh and Khataee introduced a novel non-enzymatic sensor to monitor the cholesterol level in human serum [180]. The approach aimed at a synergetic peroxidase-mimicking effect, disclosing the significance of GQDs and mixed MoS_2_ quantum dots (MoS_2_ QDs), where the intense catalytic activity of these QDs was studied on the basis of CL and the concurrent existence of this mixture was recognized by the reaction of H_2_O_2_-rhodamine B (RB) via their CL emission intensity. The authors reported a linear calibration between the CL emission intensity and H_2_O_2_ levels in the range of 1.5–460 nmol L^−1^. Nevertheless, this system was investigated to determine oxidation of cholesterol by an enzyme called cholesterol oxidase (ChOx), with regard to the enzymatic oxidation of cholesterol for the H_2_O_2_ production. This CL system attained a further enhancement by MoS_2_ nanosheets that improved the efficiency of ChOx in the cholesterol oxidation process and offered very sensitive and selective detection of cholesterol in a linear concentration (i.e., 0.08–300 µmol L^−1^), and estimated an LOD value of about 35 nmol L^−1^.

**Figure 9:**
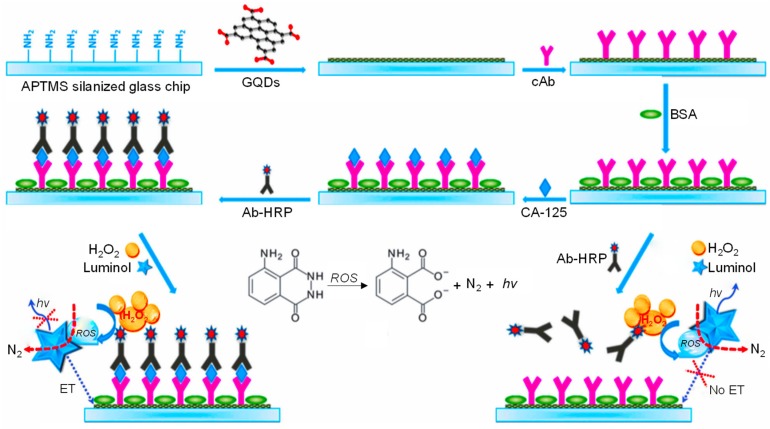
Schematic illustration of CL-based GQD sensor for CA-125 determination [181]. Reproduced by permission of The Royal Society of Chemistry.

Al-Ogaidi and team fabricated a GQD-immunosensor for quantifying the ovarian cancer biomarker CA-125 [181]. The sensor development involved the implantation of GQDs on a modified glass electrode, where the transduction of a resultant signal depended on the chemiluminescence resonance energy transfer (CRET) from the CL reagent. This CL reagent had good solubility in an aqueous media containing GQDs. The GQDs were employed as the energy acceptor, which prevented photo-bleaching occurring with organic dyes. Moreover, the GQDs facilitated the nano-surface energy transfer (NSET) mechanism, which does not need the spectral overlapping of the energy acceptor and the donor. Bio-immobilization of the capture antibody onto the GQDs-functionalized surface revealed an ability to promote high-throughput and automated sensing platforms. The fabrication strategy of this CL immuno-chip and the detection mechanism for CA-125 are depicted in Figure 9. The amino-modified glass chips were silanized with a 3-aminopropyl-trimethoxysilane (APTMS) layer, followed by the GQD coating through the electrostatic attraction of a positively charged amine group within the polydimethylsiloxane (PDMS) stencil. The specific CA-125 capture antibody (cAb) was then covalently immobilized on GQD-modified glass chip via amide chemistry. Also, bovine serum albumin (BSA) was added to clog the unreacted sites to form the GQDs–cAb chip. In the absence of CA-125 antigen, the generation of the reactive oxygen species (ROS) was catalyzed from H_2_O_2_ by virtue of an enzyme (i.e., HRP). Subsequently, this ROS could oxidize luminol to a pair of dianions, producing the electrons in an excited state. At the same time, the intensity of the emitted blue light was tracked by a fluorescence plate reader. On the other hand, in the presence of CA-125, the immunocomplex interacted with HRP and the dianion catalyzed by the HRP promoted the resonance energy transfer (RET) to the GQDs, leading to the quenched CL intensity. Thus, the CL intensity was found to be reciprocated to the CA-125 concentration. The resultant CL intensity obtained a linear correlation of CA-125 concentration, i.e., 0.1 to 600 U mL^−1^, where an LOD of 0.05 U mL^−1^ was achived in buffer. For analyzing the clinical performance of the as-prepared sensor, the CL intensity was tracked from the specimen comprising human blood plasma and buffer in 1:1 proportion, which could give an LOD of 0.08 U mL^−1^.

### 2.4. Electrochemiluminescence-Based GQD-Sensors

Electrochemiluminescence (ECL) involves electro-generation of CL, which acquires the merits of both PL and electrochemical techniques [182,183]. An ideal ECL sensor employs three electrodes; i.e., a reference electrode (RE), a counter electrode (CE) and a working electrode (WE). The electrochemical reaction that occurs on the WE leads to a particular CL reaction. In a broader range, a material is illuminated in an excited state through an electron transfer on the electrode’s surface. Consequently, the conversion of an excited state into the ground state (i.e., from unstable to stable) leads to the emission of light. Accordingly, the formation of the free radicals in ECL occurs, which are segregated into the co-reaction and quenching pathways [184,185,186]. ECL sensing techniques exhibit excellent features including high sensitivity, rapid speed of response and easy operational procedures. Moreover, they have been acutely employed for sensing numerous proteins, nucleic acids and metal ions. With reference to the fluorescence methods, ECL does not need an excitation light source, thus it avoids a scattered light background or auto-fluorescence [185,187,188]. Applied electric potential induces and controls the output signal for assuring the accuracy as well as the reproducibility of ECL. Moreover, GQDs have been witnessed as excellent ECL labeling agents for developing the GQD-sensors, especially because of their bio-compatibility and low cytotoxicity [189].

**Figure 10:**
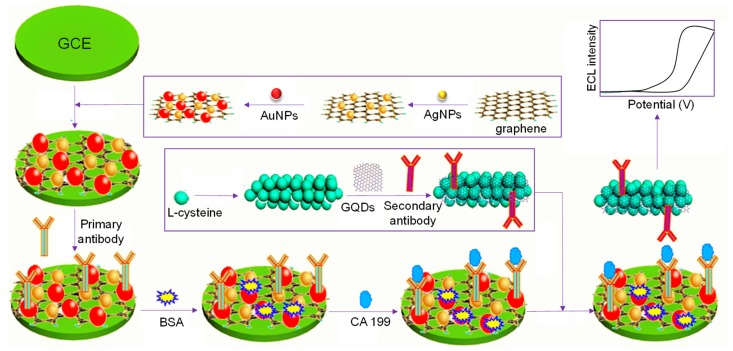
A step-wise developmental procedure of GQD-based sandwich-assay for carbohydrate antigen (CA) 199 [191].

Wu and coworkers assembled an ECL antibody sensor to determine prostate-specific antigen (PSA) involving GQDs as electrode enhancers [190]. Reinforcement of acarboxyl and NH_2_–GQDs on reduced graphene oxide (rGO)-Au/Ag NPs resulted in a considerable improvement of electrode surface as well as enhanced the conductivity that boosted the ECL signal. Nevertheless, subsequent bio-immobilization of anti-PSA antibodies dropped the ECL intensity due to the adsorbed Au/Ag. The as-formulated label-free sensor could determine PSA with an LOD of 0.29 pg mL^−1^, granting a linearity from 1 pg mL^−1^ to 10 ng mL^−1^. Another antibody sensor was proposed by Yang et al. for the detection of carbohydrate antigen 199 (CA 199) in human serum [191]. Figure 10 displays the construction steps of a sandwich assay involving the use of GQDs decorated with a porous PtPd nanochain (GQDs‒PtPd) and Au-Ag NPs-functionalized graphene (Au-Ag-GN) on GCE. By virtue of the physical and chemical characteristics offered by such hybrids, Au-Ag-GN delivered abundant sites for capturing primary antibodies and increased the conductivity, whereas GQDs‒PtPd provided a large free room for immobilizing secondary antibodies. The as-fabricated sensor could reliably quantify CA 199 molecules down to 0.96 mU mL^−1^ and achieved a linear calibration plot from 0.002 to 70 U mL^−1^. The stability studies revealed that the modified GCE could retain around 96% of its initial activity even after 50 days. This stable behavior can be ascribed to the higher affinity of GQDs‒PtPd for the secondary antibodies as well as good biocompatibility of the nanocomposites. Moreover, it was reported that this antibody sensor can be clinically employed to diagnose patients with pancreatic cancer.

Carcinoembryonic antigen (CEA) is considered as the prominent tumor biomarker for diagnosing various malignancies [192,193,194,195,196,197]. CEA is basically a glycoprotein with the size 180–200 kDa [198] and its expression and over-expression generally occur in mucosal cells and in several oncofetal tumor cells, respectively [199,200]. A rise in plasma CEA levels (i.e., >5 × 10^−9^ g mL^−1^) indicates the growth of cancerous cell [195]. In 2018, Nie et al. developed an ECL immunosensor employing GQDs for the quantification of CEA [201]. In their approach, the sensor construction held integration of GQDs in conjugation with AuNPs and electrochemically reduced graphene oxide/poly(5-formylindole) hybrids (erGO/P5FIn). Being an adequate matrix, erGO/P5FIn nanomaterial promotes the transport of ions during the occurrence of oxidation and reduction reactions, which provides a greater surface area to implant more primary anti-CEA receptors. At the same time, both AuNPs and GQDs (i.e., as labels) enhance electron transferability upon their conjugation with the secondary anti-CEA receptors. With the help of these signaling species (i.e., AuNPs/GQDs and erGO/P5FIn), the resultant sandwich-based antibody sensor could respond in the linear array from 0.1 pg mL^−1^ to 10 ng mL^−1^ CEA in a human serum, with an achievement of around 3.8 fg mL^−1^ as the detection limit.

On the basis of highly selective polydopamine (PDA) imprinted polymer and ECL properties of N‒GQDs, an emphatically engineered biosensing platform was developed [202]. This platform was intended to detect the pervasive micro-organism *E. coli*, where the sensor preparation involved direct electropolymerization of PDA and the target bacteria to develop a PDA-MIP film on the surface of GCE. Thereafter, to get rid of the bacterial template, the as-formed PDA-MIP/GCE underwent overnight incubation in a solution containing sodium dodecyl sulfate (SDS) and acetic acid. Correspondingly, N‒GQDs labeled specific polyclonal antibody was used for recognizing the target bacteria (i.e., *E. coli*). The stepwise characterization of the established electrode was performed using cyclic voltammetry (CV) and electron impedance spectroscopy (EIS). This sandwich assay was sensitive and selective for wide range of *E. coli* concentrations, i.e., from 10 to 10^7^ cfu mL^−1^. Moreover, the detection limit revealed by this sensor was 8 cfu mL^−1^, when employed for the environmental water specimen.

Lu et al. prepared graphitic structured nanocrystals of bright blue luminescent GQDs with QY of 15.5% for detecting DNA damage [203]. The research work involved the synthesis of GQDs by combining ultraviolet irradiation, thermal reduction and oxidation-cleavage, which exhibited stable ECL as well as excitation-dependent PL. During the sensor development, a cp53 ssDNA probe was conjugated with AuNPs to forge an ssDNA-AuNPs complex. The ECL signal intensity was dynamically quenched by non-covalent binding of ssDNA-AuNPs complex with the GQDs, owing to the ECL-RET taking place between AuNPs and the GQDs. Subsequent hybridization of target p53 DNA with ssDNA-AuNPs led to the formation of dsDNA-AuNPs and impairment of non-covalent binding between ds-DNA and GQDs. As a result, the ECL intensity of GQDs was restored, inducing an ECL sensing system for the target p53 ssDNA detection with an LOD and a calibration plot of 13 nM and 25–400 nM, respectively. Depending on the distinct bonding capacity for the cp53 ssDNA-coupled AuNPs and mutant target p53 ssDNA, the prepared sensor can be applied for monitoring DNA damage. The authors claimed that this method can also be employed for establishing similar methods to determine aptamer-specific target analytes or/and polymorphisms with one-nucleotide.

In 2017, Zhou et al. explored the role of GQDs as alluring ECL luminophores for the detection of glucose and H_2_O_2_, where sulfite (SO_3_^2−^) served as a reducing agent (i.e., co-reactant of GQDs’ ECL) [204]. Nevertheless, it was reported that GQDs produced stronger ECL emission (approximately 4 fold), when compared to that of spherical carbon quantum dots with SO_3_^2−^ as the co-reactant. This could be attributed to numerous surface states of GQDs. Further examination revealed that H_2_O_2_ could significantly quench GQD/SO_3_^2−^ ECL via a second-order redox reaction between H_2_O_2_ and SO_3_^2−^ under physiological pH. Given this fact, a green and facile ECL biochemical sensing system has been introduced for the determination of biomolecules like glucose and H_2_O_2_. The mechanism of the GQD/SO_3_^2−^ ECL system involved anodic generation of strongly oxidizing radicals (SO_4_^•‒^) and excited-state GQDs (GQDs*). The linear calibration plot displayed the glucose concentration from 10 to 100 µM, where an estimated LOD of 5.0 µM was achieved.

Tian and team proposed an ECL glucose sensor [205]. Prior to the sensor development, <10 nm sized GQDs were synthesized from GO sheets by treating them with photo- and sono-chemicals, where the synthetic procedure involved H_2_O_2_ as a solely used chemical compound. On applying cyclic voltammetry to GCE containing potassium persulfate (K_2_S_2_O_8_) and GQDs, a strong cathodic ECL signal was generated. Furthermore, while investigating the ECL behavior of GQDs/K_2_S_2_O_8_ co-reactant system, it was found that the ECL signal was significantly based on the reduction of GQDs and dissolved O_2_. Moreover, a product of enzymatic glucose oxidation (i.e., H_2_O_2_) quenched the signal intensity. Hence, following this mechanism, a glucose sensor based on the ECL mechanism was established by modifying GCE surface through adorning a layer of GQDs, GOx and chitosan, where the ECL intensity of glucose concentration depressed linearly from 1.2 to 120 pmol L^−1^, with an LOD of 0.3 pmol L^−1^.

Liang et al. fabricated a novel ECL-RET biosensor, exploiting the significance of GO as acceptor and GQDs as donor to monitor protein kinase activity [206]. A nanocomposite comprising GO–conjugated with anti-phosphoserine antibody (Ab-GO) was integrated onto the GQDs/phosphorylated peptide modified electrode via an immunoreaction with ATP and CK2. As a result, an ECL signal correlating with CK2 activity was generated due to the quenching of GQDs connected with GO. The ECL signal decreased in accordance with the increase in CK2 level from 0.05 to 5 U mL^−1^, which demonstrated 0.023 U mL^−1^ being the detection limit. This approach was further studied for characterizing the inhibition of CK2 activity, taking ellagic acid as a model inhibitor. Here, the ECL signal attained saturation over 0.15 µM ellagic acid and, thereby, the IC_50_ value for ellagic acid was computed to be 0.043 µM. Moreover, to study the specificity of the assay, various enzymes and proteins like glucose oxidase, alcohol dehydrogenase and BSA were experimented on. The results showed negligible variation in ECL intensity for GOx, alcohol dehydrogenase (ADH) and BSA, as compared to CK2, suggesting high selectivity of this biosensor for CK2. Henceforth, the protein kinase activity could be determined sensitively leaning on ECL-RET between GQDs and GO. The proposed ECL-RET-based biosensing strategy can be employed in clinical diagnosis and biochemical fundamental studies to screen kinase inhibition as well as to analyze CK2 activity in serum samples, implying its prominent qualitative and quantitative analytical applications.

**Figure 11:**
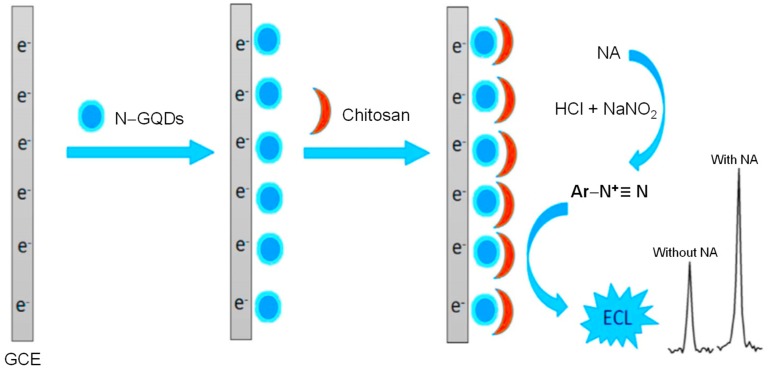
Detection strategy of nitroaniline by N‒GQDs and chitosan functionalized glassy carbon electrode (GCE) [207].

A simple ECL signal-on strategy exploring the role of N‒GQDs and chitosan was designed for detecting nitroaniline (NA) in real water samples [207]. Owing to the hydrophilicity, high water permeability and strong adhesive characteristics of chitosan, it successfully loaded the N‒GQDs to the surface of the working electrode, while N‒GQDs were used for the rapid diazotization reaction of anilines and as highly reactive agent. As shown in Figure 11, on introducing NA to the electrolyte solution containing sodium nitrite (NaNO_2_) and mineral acid (HCl), N‒GQDs/chitosan modified GCE led to an amplified ECL signal because of the diazotization event taking place. Hence, the target analyte could be detected with excellent selectivity using the chitosan/N‒GQDs ECL-sensing platform in a linear ECL signal response of NA (i.e., 0.01 to 1 μmol L^−1^). With this, the calculated value of an LOD was around 0.005 μmol L^−1^.

Yan and colleagues proposed an in situ methodology for synthesizing GQDs implanted with the nanospheres of Cu_2_O [208]. The characterization of this nanomaterial was achieved by XRD, TEM as well as X-ray photoelectron spectroscopy. Moreover, on the basis of GQDs-Cu_2_O nanospheres, a novel method for the amplification of the ECL signal of luminol system was studied, which inferred that using GQDs, the catalytic activity of Cu_2_O nanospheres was enhanced towards luminol oxidation (~3.5 times), rather than using only Cu_2_O nanospheres. Also, the ECL onset potential decreased by 130 mV. This could be ascribed to the enhanced electron transferability of GQDs. With these facts, they fabricated a model for the selective identification of a pesticide, pentachlorophenol (PCP) that strives an inhibitory effect on the ECL, which provided an LOD value of 6.6 pg mL^−1^ and could sense PCP in a dynamic linear range (i.e., 0.02 to 300 ng mL^−1^).

Depending on the ECL amplifying characteristics of CdS–GQDs nanocrystals (CdS–GQDs NCs), an ultrasensitive ECL sensor was designed by Liu et al. for the recognition of PCP contamination in water [209]. Because of the existing doped GQDs, the ensuing CdS–GQDs NCs not only displayed five times increase in ECL sensor response than plain CdS NCs, but also resulted in a negative shift in ECL onset potential by 80 mV. Accounting for the selective inhibitory effect of PCP on the ECL intensity of GQDs–CdS NCs, this facile method could achieve an LOD of 3 pg mL^−1^ and determined PCP concentration in a dynamic linear array of 0.01–500 ng mL^−1^ with good reproducibility as well as long-term stability.

Another ECL method for sensing PCP in water was introduced by Du and colleagues [210]. In this approach, preparation of N‒GQDs was conducted via a simple hydrothermal technique involving the dissection of nitrogen-doped graphene, and thereafter N-doped GQDs were uniformly functionalized onto the graphene oxide (GO) surface to obtain NGQDs-GO nanocomposites. The as-synthesized N‒GQDs with GO as the immobilization support possessed excellent ECL behavior i.e., a 3.8-fold higher ECL response than simply using N‒GQDs, and also led to a decreased ECL onset potential by 200 mV. This ECL sensor could identify PCP concentration in a linear range, i.e., from 0.1 to 10 pg mL^−1^. Here, the detection limit was quantified down to 0.03 pg mL^−1^. When we compared its features with both of the ECL sensors reviewed above for PCP, it was observed that this sensor exhibited 100- to 220-fold lower detection limits. Such an ultra-sensitivity can only be achieved because of the presence of GO apart from GQDs.

Dong et al. reported an ECL system for the selective determination of lead (II) ions (Pb^2+^) using single-layer GQDs and L-cysteine (L-Cys) [211]. This L-Cys/GQDs co-reactant system generated a strong cathodic ECL signal depending on some critical parameters such as oxidation of L-Cys, reduction of GQDs and the presence of dissolved O_2_. Besides, it was found that Pb^2+^ could quench the ECL signal of the L-Cys/GQDs system. The authors reported that this methodology can deliver a quick and reliable strategy for Pb^2+^, with an LOD of 70 × 10^−9^ M and a wider linear response of 100 nM–10 μM.

**Figure 12:**
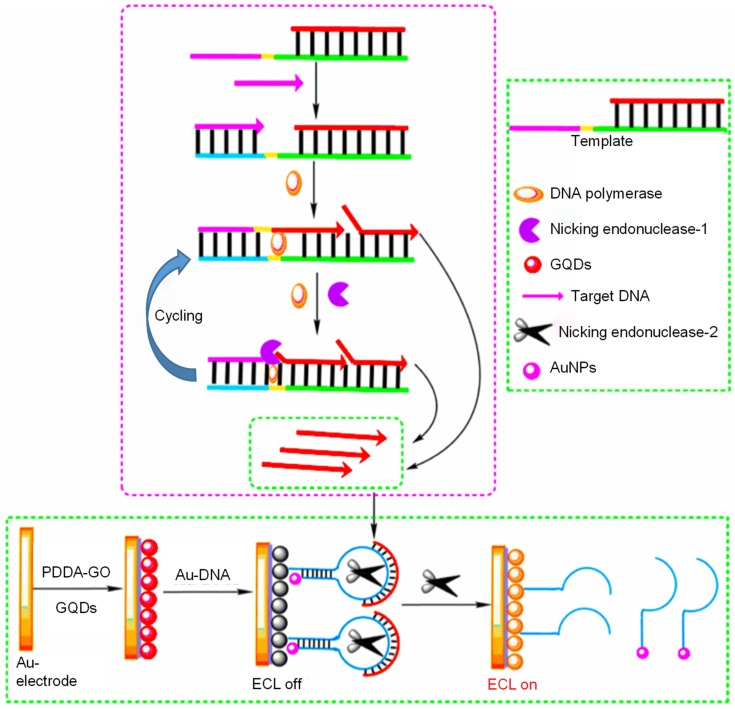
The mechanism involved in detecting DNA by GQD electrochemiluminescence (ECL) assembled with the multiple cycling amplification method [212].

In 2019, Jie et al. developed an enhanced ECL approach for the DNA analysis. As shown in Figure 12, this method was based on the ECL activity of GQDs associated with a multiple cycling amplification strategy [212]. The uniform GQDs were synthesized and then immobilized on the electrode surface by graphene oxide/poly-diallyldimethylammonium chloride (GO/PDDA) hybrid material that amplified the ECL signal and stability of GQDs. This signal-on ECL system for detecting DNA employed ECL quenching from AuNPs to GQDs coupled with an endonuclease-aided cyclic amplification strategy. Moreover, this biosensor could detect target DNA in a wide linear range (i.e., 1.0 × 10^−12^ M–1.0 × 10^−6^ M), with the quantification limit of 0.1 × 10^−12^ M and can be further expanded for other biosensing applications, particularly in clinical diagnosis.

A mono-luminophor ECL sensor using N‒GQDs was established for the ratiometric sensing of Co^2+^ ions in real water samples [213]. Dissolved O_2_ converted to O_2_^−^ and HO_2_^−^ aided in generating anodic and cathodic ECL signals of N‒GQDs, while Co^2+^ ions could clearly enhance the ECL intensity at the anode through the catalytic performance occurring during the transitional period, and could quench the ECL intensity at the cathode due to its considerable removal from N‒GQDs*. Thus, the Co^2+^ ions were determined by analyzing the ratiometric excitation potentials. With these findings, a dual-potential label-free ratiometric ECL sensing system for Co^2+^ ions was manufactured with high sensitivity and selectivity against other interfering metal ions such as Cu^2+^, Fe^2+^, Hg^2+^ and Ni^2+^ ions. The as-fabricated ECL-sensor provided a linear signal response for Co^2+^ ions (i.e., 0.001–0.07 M), with an LOD of 0.2 mM.

Lu et al. designed an ECL aptasensor for determining adenosine triphosphate (ATP) [189]. The study involved the hydrothermal preparation of water-soluble GQDs through disintegration and exfoliation treatments with GO. The as-synthesized GQDs presented an intense blue light through a UV irradiation (~365 nm) that exhibited a PL property. An ECL emission with a strong light was generated at anode (Ag/AgCl vs. ~0.4 V). Furthermore, SiO_2_ nanospheres were used as signaling species and, therefore, GQDs/SiO_2_ ECL labels were developed for an ultrasensitive ECL aptasensor, which could detect ATP in a near-linear relationship between 5 p mol L^−1^ to 5 n mol L^−1^. This aptasensor achieved an LOD of 1.5 p mol L^−1^. Further examples of ECL-based GQD-sensors are listed in Table 3.

### 2.5. Fluorescence Resonance Energy Transfer (FRET)-Based GQD Sensors

Technically, FRET is a phenomenon that transfers radiation-free energy from one fluorophore to another, i.e., from a donor to an acceptor. In a broader range, the energy from the excitation light source is absorbed by the donor and non-radioactively transferred to the acceptor, resulting in an emission of fluorescence. This distance-dependent feature is employed for the examination of structural variations during the bio-recognition events [223]. FRET-dependent sensors have been employed to monitor the cell-dynamics in heterogeneous cellular populations as well as to study on-site single-cell concentration. In spite of their divergent biomedical applications, such sensors still face some challenges. Hence, there is a huge demand for the enhanced sensitivity as well as for the higher fluorescence resolution of FRET biosensors [11].

**Figure 13:**
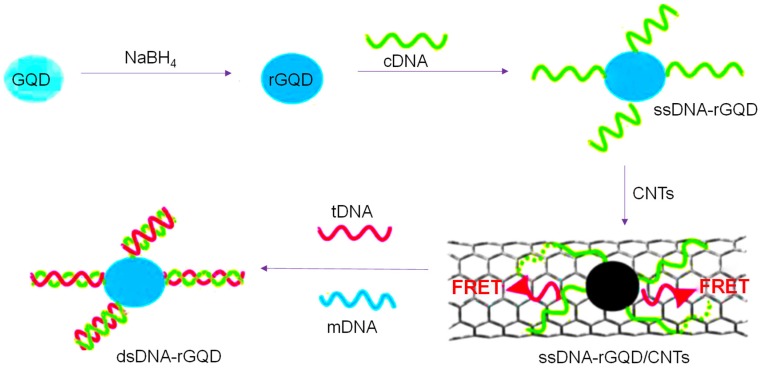
DNA detection strategy of GQD-based fluorescence resonance energy transfer (FRET) nanosensor [224].

As represented in Figure 13, Qian and co-workers reported an ultrasensitive DNA nanosensor based on FRET between bio-compatible GQDs and CNTs for the quantitative analysis of target DNA, [224,225]. Herein, the fabrication strategy was implemented by considering: (a) the base-coupling particularity of DNA, (b) distinct FRET between CNTs and DNA, (c) strong fluorescence and excellent biocompatibility of GQDs. In this approach, oxidized CNTs and GQDs with QY up to 0.21 were synthesized as a capable quenching agent and fluorophore of a DNA probe, respectively. FRET between oxidized CNTs and a GQD-labelled probe was attained by the virtue of their self-assembly via π–π stacking. Typical “on-off-on” fluorescence was induced from fluorescence quenching (30 min) due to FRET between GQDs and CNTs as well as consequent fluorescence recovery (30 min) owing to the released probe of free double stranded (ds) DNA. The as-developed nanosensor could differentiate mismatched and complementary nucleic acid sequences with high sensitivity and acceptable reproducibility. The detection strategy based on this nanosensor acquired a wide linear response and an LOD of 1.5–133 nM and 0.4 nM, respectively.

Acid phosphatase (ACP), a prevalent digestive enzyme, is highly specific for hydrolyzing the phosphate esters under an acidic environment [226]. Generally, the human ACP level is low in mammalian cells, but prostate cancer, colon cancer, Gaucher disease, renal disorders, diseases related to veins and bones are usually tailgated by changes in the ACP concentrations [227,228]. Clinically, the ACP activity is measured to diagnose cancers and to monitor cell viability. Na et al. developed a new and efficient fluorescence method for selectively and sensitively determining ACP activity [229]. This bio-sensing system was constructed by linking GQDs to Nile red (NR) through a complex of β-Cyclodextrin/lecithin (β-CD/lecithin). The coexistence of β-CD/lecithin as a linker molecule, would bring a pair of NR–GQDs together via hydrophobic as well as electrostatic interactions, resulting in FRET and, thereby, fluorescence enhancement and quenching of an acceptor and donor (i.e., NR and GQDs), respectively. The ACP could hydrolyze lecithin into 2 fragments, which could separate a pair of NR–GQDs. This FRET sensor was clinically applied for ACP imaging in prostate cancer cells (PC-3M), which could detect very low concentration of ACP with an LOD of 28 µU mL^−1^. Moreover, the authors reported that the tolerable concentration ratios of other interfering compounds to 100 µU mL^−1^ ACP was 10 times higher for ATP, ALP, Try, Lys and GSH, suggesting the as-developed strategy is quite selective for ACP.

A homogeneous assay for quantifying DNA in the case of transgenic soybean, based on FRET, was exemplified for cauliflower-associated mosaic virus 35s (CaMV35S) [230]. The assay involved DNA hybridization with a probe functionalized by AgNPs and N‒GQDs (i.e., acceptor-donor pairs). The highly conducive FRET and distinct features of N–GQDs revealed an efficient FRET detection platform for labeling DNA. Upon the specific target DNA (tDNA) recognition, the FRET between AgNPs and NGQDs facilitated fluorescence quenching for tDNA detection. The as-developed homogeneous FRET method exhibited a broad linear relationship between 0.1 and 500 × 10^−9^ M. The interpretation of the low quantification limit for the detection of the CaMV35S promoter was reported as 0.03 nM. This biosensor was highly specific for tDNA, with long-term stability and excellent intra-assay precision. Moreover, this assay was applied to determine the real sample of transgenic soybean and was further validated by polymerase chain reaction (PCR), indicating its feasibility to determine genetically modified organisms for routine applications.

In 2018, Kong and co-workers engineered FRET based immunosensor for the quantification of von Willebrand factor (vWF), a multimeric plasma glycoprotein. Initially, they synthesized Ag_core_@Au_shell_ nanoparticles (Ag@AuNPs) and GQDs that were then characterized by X-ray photoelectron spectra and TEM, respectively [231]. During the assay development, vWF antibody was immobilized on Ag@AuNPs to obtain the Ag@Au-antibody complex. This nanocomplex could readily quench the fluorescence of GQDs by virtue of FRET. The immunoreaction occurring between Ag@Au-antibody and vWF antigen led to decreased FRET efficiency as well as the fluorescence recovery of GQDs. The variation in the resultant fluorescence emission was directly co-related with the logarithmic vWF concentration (0.1 pg mL^−1^‒10 ng mL^−1^), with the estimated LOD of 30 fg mL^−1^. This biosensor was applied to study the vWF release pattern and the oxidation-injury profile of vascular endothelial cells, where the experimental values suggested that the vWF content in the growth medium was elevated and the cell injury was intensified due to the increased cellular contact with H_2_O_2_.

Poon et al. fabricated an efficient nanoprobe based on FRET for quantifying trypsin [232]. In this approach, GQDs and CMR2 (i.e., coumarin derivative) were employed as a donor and as an acceptor, respectively. BSA was utilized as a linker as well as a fluorescence enhancer of GQDs for the FRET pair. During the presence of trypsin, this model would be devastated due to the digestion of BSA by trypsin, resulting into the regeneration of an emission peak of the donor and an increased ratio of the emission peak of the donor to the acceptor. On the basis of these ratiometric values, trypsin could be quantitatively analyzed, where an estimated LOD was reported to be 0.7 µg mL^−1^ (i.e., 0.008 times the mean value for trypsin concentration found in urine of the individuals suffering from acute pancreatitis).

The testing of intracellular GSH level in human serum has garnered meticulous attention due to its vital role in several human pathologies. The development of a ‘turn off−on’ fluorescence nanosensing system exploiting the attributes of MnO_2_ nanosheets and GQDs was reported to quantify GSH levels [233]. In the study, MnO_2_ nanosheets could quench GQDs’ fluorescence through FRET. Nonetheless, GSH could convert MnO_2_ nanosheets into Mn^2+^ via a reduction reaction and liberate GQDs, resulting in fluorescence restoration. The MnO_2_ nanosheets could perform as a nano-quencher as well as a GSH recognizer in the biosensor. The as-prepared sensing device could probe GSH concentrations, ranging from 0.5 to 10 μmol L^−1^ and a minimum detectable concentration of 150 nmol L^−1^. Moreover, the GQDs−MnO_2_ nanoprobe was highly selective for GSH against interfering biomolecules as well as electrolytes.

In 2018, a FRET-sensor using AuNPs as well as GQDs was proposed to evaluate glutathione reductase (GR) activity in human [234]. The sensing mechanism followed several facts, which included: (a) quenching of GQDs’ fluorescence emission intensity by AuNPs via FRET; (b) the role of reduced GSH in preventing AuNPs from agglomeration and enlargement of the inter-particle distance between AuNPs and GQDs that generates the fluorescent signal recovery; (c) weaker affinity of AuNPs towards oxidized glutathione (GSSG) than towards GSH; (4) catalytic performance of GR to convert GSSG into GSH in the existence of β-nicotinamide adenine dinucleotide 2’-phosphate hydrate (NADPH). With such findings, a ‘turn-on’ bioassay could monitor GR activity in a linear fashion of 0.0050−0.13 mU mL^−1^ and provided an LOD of 0.0050 mU mL^−1^. This AuNPs−GQDs bioassay was also validated for checking the inhibition activity of GR, with 1,3-bis(2-chloroethyl)-1-nitrosourea (BCNU) being an illustrative molecule.

**Figure 14:**
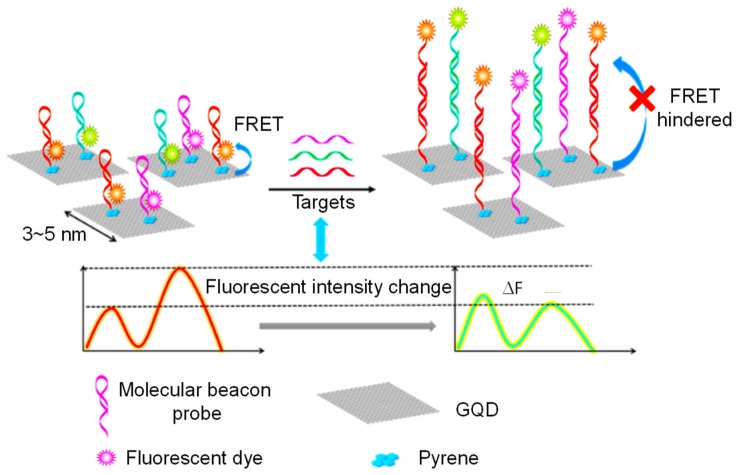
Biosensing system based on FRET change between GQDs and pyrene-modified molecular beacon probes (py-MBs) for determining miRNAs [236].

Sun and team proposed a biosensor for the diagnosis of cardiovascular diseases by considering miRNA-34a as a model analyte [235]. The strategy worked on the principle of “off-on” through the use of a GQDs probe coupled with gold nanoflower (AuNF). The authors reported that, when AuNF and GQDs are at a distance of 4 nm and when the probe is coupled with a target miRNA, they result in light-up sensitivity as well as fluorescence quenching efficiency and promote DNA circuit strategy. The target miRNA-34a could be sensed to 0.1 fM with a wide linear response ranging from 0.4 to 4 fM. Another biosensing system for determining miRNAs using GQDs as well as pyrene-modified molecular beacon probes (py-MBs) was demonstrated by Zhang et al. [236]. As portrayed in Figure 14, pyrene was added for the specific induction of FRET between fluorescent dyes labeled with py-MBs and GQDs through π−π stacking. The variation in fluorescent intensity could develop a novel signal mechanism for determining the target miRNAs in a wide range (0.1–200 nM) and could give an LOD value of 0.1 nM. The articulately developed FRET biosensor showed a new direction for detecting small nucleic acids with multiplex detectability. Furthermore, the authors claimed that this biosensor can be clinically convenient for diagnosing several cancer-types in their early stage.

Fan and group conducted chemical synthesis of multicolored fluorescent GQDs from GO to develop a FRET-based sensing platform for 2,4,6-trinitrotoluene (TNT) [237]. Herein, the GQDs could bind TNT through π‒π stacking interaction between the aromatic rings and GQDs, which resulted into a decreased fluorescence intensity from GQDs to the non-radiative TNT (i.e., from donor to an acceptor). The unfunctionalized GQDs could quantify TNT in a broad linear range from 4.95 × 10^−4^ to 1.82 × 10^−1^ g L^−1^, with the detection limit of 0.495 ppm (i.e., 2.2 mM) by using just a milliliter of GQDs solution.

Shi and group conducted a FRET-based biosensing experiment, exploring the role of AuNPs as well as GQDs for *Staphylococcus aureus* (*S. aureus*) specific gene sequence determination [238]. Herein, reporter probes were fused on AuNPs and capture probes were implanted on GQDs, where these nanomaterials acted as the energy acceptor and donor pairs, accordingly. To induce the FRET effect, co-hybridization of the target oligonucleotides was carried out with reporter and capture probes that could bring AuNPs and GQDs in close proximity. The initial and final fluorescence signal responses were controlled, where the resulting quenching efficacy could attain almost 87% using only 100 nM of the target oligonucleotides. An LOD could be achieved down to 1 nM for the target bacterial gene identification. The selectivity studies with both mono-base and dual-base mismatched oligonucleotides has shown the efficiency of this sensor in the real world.

Bhatnagar et al. fabricated a FRET-immunosensor based on amine functionalized GQDs to diagnose myocardial infarction (MI) at its early stage [239]. In the research work, the anti-cTnI antibodies were covalently attached to GQDs via 1-ethyl-3-(3-dimethylaminopropyl)-carbodiimide (EDC) and N-hydroxysuccinimide (NHS). Characterization of this anti-cTnI/GQDs conjugate was carried out using techniques like zeta potential UV–Vis spectroscopy and field-emission scanning electron microscopy (FE-SEM). A detection efficiency associated with this immunosensor was examined in accordance with the photoluminescence and variation in photon count of GQDs, which depended on the recognition event between the target cTnI and anti-cTnI antibody. The authors reported that this sensor displayed a minimum response to the other interfering antigens and provided a wide linear relationship (i.e., 0.001–1000 ng mL^−1^), with an LOD of 0.192 pg mL^−1^. This antibody sensor could quantify cardiac troponin (cTnI) biomarker within just a couple of minutes (precisely: 10 min).

An FRET-biosensor was constructed for detecting DA using poly-dopamine (pDA)-implanted GQDs [106]. DA could be self-polymerized to pDA for developing a thin layer with GQDs in an alkaline environment that resulted in fluorescence quenching due to FRET. Such a sensing platform could respond sensitively to DA in a broad linearity, ranging from 0.01 µM to 300 µM, with an LOD of 8 nM in human serum.

## 3. Electrochemical GQD Sensors in Biomedical Diagnostics

Electrochemical sensors acquire a substantial ranking among commercially available sensors and gained popularity in biomedicine [240,241,242]. This sensor type can act as a miniaturized device for point-of-care testing (POCT) [243,244]. They are built upon a WE (i.e., transducer and recognition system), a CE and a RE for computing the electrical signal response [245,246]. Among all the GQD-based electrochemical sensors working on different kinds of receptors (i.e., antibody sensors, MIP-sensors, DNA sensors, aptasensors and enzyme-based sensors), an antibody-sensing system has been widely studied [79]. It is often known as an immunosensor, because its function is solely influenced by the immunoreaction [247,248]. Such sensing platforms involve antibodies exhibiting excellent affinity for their corresponding target biomolecules [11,79,249]. Taking immunoreactions into an account, typical immunosensors can work on several assay-modes. (i.e., competitive, direct, sandwich or indirect modes) [250,251,252,253]. Such assay formats work on the unique mechanisms and usually entail the events mentioned below [11,79]:

1. Implantation of the desired biomolecule.
2. Blockage of the un-reacted sites.
3. Determination of the target biomolecule.

**Figure 15:**
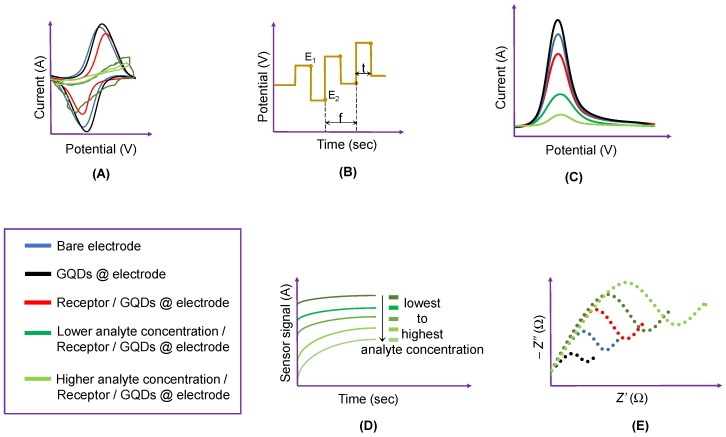
(**A**) Cyclic voltammetry (CV): potential vs. current profile with reference to a GQD-implanted electrode surface, where its electrochemical response changes according to the specific bio-recognition event. (**B**) Square wave voltammetry (SWV): potential vs. time profile, where t: time required for each pulse, f: frequency of each pulse, E_1_ and E_2_: initial and final pulse, respectively. (**C**) Target biomolecule detection by GQD-coated electrode based on SWV, where the current changes in direct correlation with an occupancy of a specific receptor. (**D**) Ideal curves observed with amperometric measurements for varied concentrations of target biomolecules. (**E**) Electron impedance spectroscopy (EIS): variation in impedance due to the modification of electrode surface and subsequent introduction of a target analyte (i.e., Nyquist plot). Adapted from Mansuriya and Altintas [79].

A chemical or biological reaction can be transduced into an electrical response by several forms of electrochemical techniques, i.e., using voltammetry, potentiometry, impedimetry, conductometry or/and amperometry. However, conductometric and potentiometric techniques are not yet actively employed in developing electrochemical GQD sensors, due to their several limitations such as low-sensitivity, less-stability, less-specificity and inaccuracy [254]. Therefore, in this section, we discuss the other three techniques in detail. Figure 15 shows the corresponding plots for voltammetric, amperometric and impedimetric measurements, which are briefly explained in the ensuing sections.

### 3.1. Voltammetric GQD-Sensors

Voltammetric sensors employ the applied potential (*E*) to the surface of an electrode and monitor the generated flow of current (*i*), where the amount of electroactive species is varied with respect to time through redox reaction. The benefits of voltammetric techniques reside in their marvelous sensitivity, quick analysis, concurrent detection of different analytes, generation of different potential waveforms, small current measurements and determination of kinetic parameters. Voltammetric biosensing methods can be based on polarographic, stripping voltammetric, linear scan or sweep voltammetric, differential pulse voltammetric (DPV), normal pulse voltammetric (NPV) or CV measurements [255,256,257,258,259].

While controlling the current in case of CV, the application of potential at a WE is swerved both ways, i.e., reverse as well as forward (Figure 15A). Based on the desired analytical measurements, a partial cycle, an entire cycle, or multiple cycles can be conducted [257]. In the case of NPV, the current is computed after every pulse. Generally, each pulse endures for about 1 to 100 milliseconds and the interval between each pulses is 0.1 to 5 s [255,256]. DPV is more or less similar to NPV, which also involves a potential scan with a series of pulses. On the other hand, it is also distinguishable, because each potential pulse is well defined in case of DPV and they are of small amplitude (10 to 100 mV). Here, the current is computed two times for every pulse i.e., initially as well as finally [255,256]. The SWV excitation response constitutes a defined symmetry like a ‘staircase pattern’ (Figure 15B). SWV is a widely used method, because its advantages lie in providing excellent sensitivity and increased signal/noise ratio [258,259]. Moreover, the typical SWV peak right from the electrode functionalization to the target detection is displayed in Figure 15C. The advantages of pulse strategies are that the decay rates are different, where the faradic current is too high as compared to the capacitive current. This faradaic/capacitative current ratio leads to the achievement of an LOD, which makes such techniques eligible for biosensing wide range of biomolecules [260]. Moreover, SWV and pulse methods are reported to be more extensively employed than other techniques for the analytical purposes [261,262].

Ensafi et al. fabricated a MIP sensor for quantifying metronidazole (MNZ) [263]. Initially, MIPs were deposited on GQDs that were produced by the sol–gel–sol method. Thereafter, they were cast on GCE functionalized with graphene nanoplatelets (GNPs). Due to the combined additive effect of GNPs and MIPs@GQDs, the sensor showed noticeably improved electro-catalytic activity. The electrochemical characterization of the modified GCE was successfully achieved by CV, DPV and EIS techniques. This MIP-based electrochemical sensor could display two distinct linear ranges in human blood plasma, i.e., from 0.005 to 0.75 µmol L^−1^ and 0.75 to 10.0 µmol L^−1^, and could offer a lower quantification limit of 0.52 nmol L^−1^ for MNZ.

Hatamluyi and Es’haghi developed a MIP based electrochemical sensor to identify 6-mercaptopurine (6-MP) [264]. Herein, QDs exhibiting ZnO core covered on a shell of graphene (i.e., ZnO@GQDs) were prepared, which were then implanted on the pencil graphite electrode (PGE) grafted with a sol–gel binder and electro-polymerized with polypyrrole (PPy). The as-prepared GQDs were characterized by energy dispersive X-ray (EDX), FE-SEM, FT-IR and XRD, while the sensor surface had been characterized via CV and EIS. By using DPV, the electrochemical detection of 6-MP could be possible in a broad linear range of 0.01 to 700.0 µM, with an LOD of 5.72 nM.

Yola and Atar constructed a MIP-based sensor for analyzing the serotonin (SER) levels in urine [265]. Herein, GQDs were fused with 2D hexagonal boron nitride (2D-hBN) nanosheets, incorporated to the GCE surface and characterized by using TEM, XRD, CV, EIS, SEM, and X-ray photoelectron spectroscopy (XPS). This MIP-based voltammetric sensor could sense SER from 1.0 pM to 10 nM. Moreover, this sensor attained an LOD of 0.2 pM with high specificity for SER against several interfering compounds like norepinephrine, dopamine and tryptophan. In 2019, Roushani et al. also established a MIP-based sensor, but to check the sotalol (SOT) level in human blood serum as well as in pharmaceutical tablets [266]. This sensor preparation involved the functionalization of SPCE with thiol doped GQDs (GQD–SH) and AuNPs, where the modified sensor could quantify SOT in the linear calibration range from 0.1 to 250 μM, with an LOD of 0.035 μM. Having acted as a sublayer in the nanocomposite mixture, GQD–SH offered a large surface area for the further sequential immobilization of SPCE. Moreover, AuNPs were implanted on to the SH groups of GQDs via an Au–S bond formation, resulting in the increased electronic transfer rate, accelerated electrochemical signal response, as well as enhanced adsorption of the SOT on SPCE.

**Figure 16:**
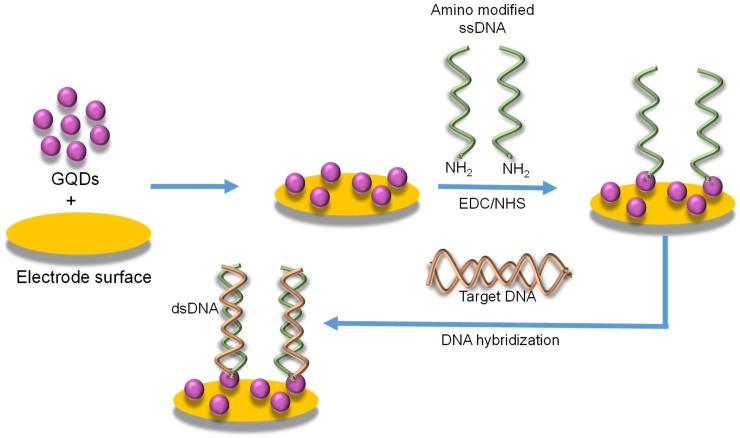
An example of a GQD-based electrochemical DNA biosensor.

An electrochemical DNA sensor was introduced by using peroxidase-like magnetic ZnFe_2_O_4_–and GQDs hybrid nanomaterial [267]. This hybrid nanomaterial was prepared using a photo-Fenton reaction that was employed as an enzyme-mimicking label and also as a carrier for labeling complementary ssDNA. The modification of ZnFe_2_O_4_/GQDs/GCE was subsequently accomplished by a self-assembly approach between aminated graphene and Pd nanowires, which could accelerate the electron transfer efficacy and immobilize a large amount of capture ssDNA. In the study, thionine was chosen as an electron mediator and, with the assistance of an enhanced electro-catalytic performance exhibited by ZnFe_2_O_4_/GQDs, a large current was generated to reduce H_2_O_2_ for signal amplification. This sensor could show a remarkable linear range from 1 × 10^−16^ to 5 × 10^−9^ M of target DNA, with the detection limit of 6.2 × 10^−17^ M. Joshi and Waghmode used GQDs for the sensitive detection of p16, a tumor-suppressor gene causing pancreatic cancer [268]. In this work, an on-chip sensing system was exploited for p16 quantification through various electrochemical methods like DPV and EIS and were characterized by employing several other techniques such as TEM, SEM and UV–Vis spectroscopy to develop a facile DNA hybridization sensor (Figure 16) for the early diagnosis of pancreatic cancer, which possessed an LOD of 0.10 pM.

In 2019, Mahmoudi-Moghaddam et al. provided an electrochemical strategy for topotecan detection by investigating topotecan–DNA interaction on carbon paste electrode (CPE) [269]. The biosensor was constructed by functionalizing a CPE with ionic liquid (IL), GQDs and double-stranded DNA (ds-DNA). Such a sensing platform could deliver a quantification limit down to 0.1 μM for topotecan through DPV technique in a wide concentration range from 0.35 to 100.0 μM. This DNA biosensor can be clinically applied to sense topotecan in human blood serum as well as in urine.

In 2017, Arvand and Hemmati exemplified an electrochemical-sensing technique to monitor the progesterone (P4) levels in human blood [270]. In this strategy, GQDs, Fe_3_O_4_ NPs and functionalized multi–walled CNTs were decorated onto the GCE. The synthesis of GQDs was conducted via a bottom–up approach through the citric acid carbonization and disintegration of the carbonized species into an alkaline media. The as-modified GCE surface had been characterized by voltammetric techniques, FT-IR, TEM, XRD and UV–vis spectroscopy. Electrochemical investigations implied some benefits of this sensor, which included larger surface area, abundant reactive sites and better electro-catalytic activity to oxidize P4. The authors claimed that this sensor could be highly specific for P4 and had responded efficiently to P4 over a wide dynamic linear range from 0.01 to 3.0 μM, with an achievement of 2.18 nM as the detection limit.

An enzyme-based sensor had been demonstrated by Vasilescu et al., for calculating the total polyphenolic content in red wine [271]. Herein, a nanocomposite comprising MoS_2_ nanoflakes and GQDs was used as a base to immobilize an enzyme, laccase, on SPCE. Once the SPCE surface was functionalized by MoS_2_ nanoflakes and GQDs, an increased conductivity of the SPCE was observed. Moreover, this modified SPCE could deliver a biocompatible matrix for an effective immobilization of laccase. The laccase biosensor could measure caffeic acid in the range of 0.38 to100 µM, with an LOD of 0.32 µM.

A two-step biomolecule-mediated self-assembly approach to establish ternary GQD-peptide nanofibers (PNF)-GO nanohybrid material was reported for sensing H_2_O_2_ [272]. At first, PNFs were used as templates to construct the N‒GQDs nanowires by employing a one-step self-assembly technique and then, binary GQD-PNF nanohybrids were produced, which were assisted by GO nanosheets to form 2D ternary GQD-PNF-GO nanohybrid material. The principle behind the π–π interaction occurring between the materials residing within 2D ternary GQD-PNF-GO nanohybrids was examined by TEM and atomic force microscopy (AFM). This electrochemical H_2_O_2_-sensor could perform well in a dynamic linear range from 10 μM to 7.2 mM, with an LOD of 0.055 × μM. Also in 2017, an electrochemical sensor was presented for testing H_2_Q in environmental water samples [273]. The sensor was hybridized with metal oxide and GQDs on the basis of their electrochemical features. Herein, Co_3_O_4_-histidine-modified GQDs were synthesized to generate Co-HiS‒GQD conjugate, following their thermal annealing at 350 °C in air to obtain Co_3_O_4_-HiS‒GQD. The resulting hybrid nanomaterial could provide a 3D structure enriched with porous architecture. On graphene sheets, the development of Co_3_O_4_ behaved in a similar fashion as that of the electrochemical contacts between Co_3_O_4_ and HiS‒GQDs, which facilitated a rapid electron and energy transfer between HiS‒GQDs and Co_3_O_4_ within the hybrid nanomaterial. Using DPV, the voltammetric peak current of H_2_Q revealed an increased linear relationship (i.e., from 2 × 10^−9^ to 8 × 10^−4^ M). The authors reported an LOD value of 8.2 × 10^−10^ M for H_2_Q.

In a work based on the electrochemical detection of L-cysteine, a polypyrrole (PPy) and Prussian Blue (PB)/GQD nanomaterial was incorporated to modify a graphite felt (GF) electrode [274]. The GQDs were synthesized by ultrasonication via a carbonization reaction of citric acid and grafted on the GF surface that induced the synthesis of PB through a simultaneous oxidation and reduction between [Fe(CN)_6_]^3−^ and Fe^3+^. The electropolymerized PPy layer led to the enhanced stability of a PPy/PB/GQDs@GF electrode, which was then characterized by infrared (IR), XRD, TEM, SEM and electrochemical methods. The sensor could efficiently oxidize L-cysteine by virtue of its electro-catalytic performance, ranging from 0.2 μmol L^−1^ to 1.0 mmol L^−1^, with an LOD of 0.15 μmol L^−1^ and showed a quite negligible response to the other interfering compounds.

**Figure 17:**
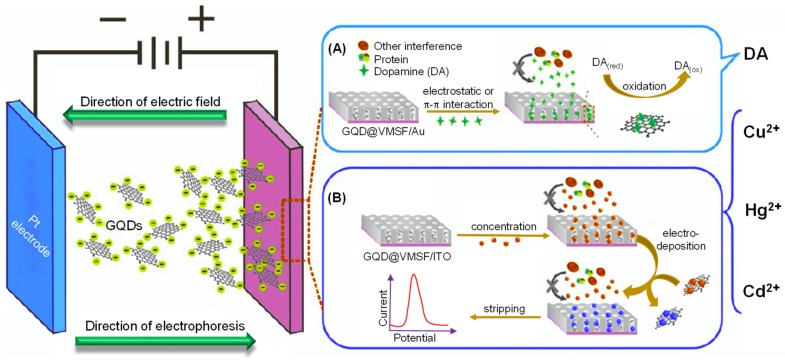
(**A**) Casting procedure of GQD@VMSF (vertically-oriented mesoporous silica-nanochannel film) modified electrode via electrophoresis. (**B**) Quantification of Cu^2+^, Hg^2+^ and Cd^2+^ ions as well as dopamine. Adapted from Lu et al. [275].

In 2018, Lu et al. fabricated a voltammetric sensing device comprising nanochannel-confined GQDs to distinguish and quantify several metal ions (i.e., Hg^2+^, Cu^2+^ and Cd^2+^ ions) from environmental and food samples as well as dopamine in human blood [275]. In this research, a vertically-oriented mesoporous silica-nanochannel film (VMSF) was terminated on the electrode surface, providing an excellent antifouling and anti-interfering characteristics to the electrode by steric exclusion and electrostatic repulsion mechanisms. The as-prepared GQDs, rendering multi-functionality, were confined in the nanochannels of VMSF via electrophoresis, and served as the signal amplifier as well as the recognition element. As illustrated in Figure 17, this versatile electrochemical method could quantify Hg^2+^, Cu^2+^ and Cd^2+^ ions with the respective LOD values of 9.8 pM, 8.3 pM, and 4.3 nM. Moreover, it can also detect dopamine with a minimum concentration of 120 nM.

Lie et al. constructed an electrochemical sensor for dopamine [276]. The study aimed to immobilize GQDs on GCE via a covalent self-assembled approach, which was subsequently examined by several characterization techniques like TEM, FT-IR, AFM, CV, EIS and DPV. This electrochemical GQD-sensor could respond to dopamine in a linear range from 1 μM to 0.15 mM, with the quantification limit of 115 nM. Besides, the authors stated that this sensor could be highly specific to dopamine, giving a negligible response to the other potent interfering molecules such as ascorbic and uric acids.

An electrochemical agricultural analysis of L-DOPA was examined by functionalizing the surface of GCE with GQDs, magnetic NPs and multi-walled carbon nanotubes (MWCNTs) [277]. Here, the GQDs were prepared by pyrolyzing citric acid and all these nanomaterials underwent characterization by TEM, XRD and FT-IR methods. With the help of DPV, this biosensor could deliver sensitive and selective identification of L-DOPA, ranging from 3.0 to 400 μmol L^−1^, with an LOD of 14.3 nmol L^−1^. In the same year, an electrochemical experiment was carried out to analyze a drug, doxorubicin hydrochloride (DOX-HCl), where the GQDs were synthesized by a bottom-up approach and then, tightly absorbed on the GCE surface [278]. The CV profile suggested that the GQD-coated GCE could conspicuously increase the electro-catalytic behavior toward the oxidation of DOX-HCl, while DPV was also employed for confirming the DOX-HCl response that ranged in a broad linear spectrum from 0.018 to 3.6 μM, with an LOD value of 0.016 μM in human plasma.

Selecting GQDs and AgNPs as GCE modifiers, an electrochemical concept for the concurrent oxidation of guanine and adenine was studied, where the sensing platform could not only just quantify them, but also exhibited an ability to discriminate these purine bases [279]. The GCE functionalized with GQDs/AgNPs could reveal a decrement in anodic peak overpotentials and an increment in anodic peak currents. At the same time, the modified GCE surface was enlarged nearly 22 fold as compared to the bare surface, suggesting the significance of using these nanomaterials. The linear calibration plots for guanine and adenine were found in the range from 0.015 to 430 μM and 0.015 to 390 μM, respectively. Moreover, the corresponding LOD values were computed as 10 nM and 12 nM, respectively.

Hasanpour and coworkers casted a voltammetric sensor for evaluating dobutamine (DB) levels in human serum, which is a catecholamine drug to treat coronary heart disease and cardiogenic shock [280]. Here, N–GQDs were sourced from citric acid and urea and then, nickel manganese ilmenite-type (NiMnO_3_) was synthesized in the presence of N–GQDs using a one-step hydrothermal route. This nano-hybrid material was characterized by XRD, SEM as well as FT-IR and functionalized on CPE that enabled the electron transfer rate as well as the catalytic activity for electro-oxidation of DB. The oxidation aspect of DB was approved by CV, where a linear response of DB concentration ranged between 0.08–40.0 µM, with an LOD of 0.02 µM.

**Figure 18:**
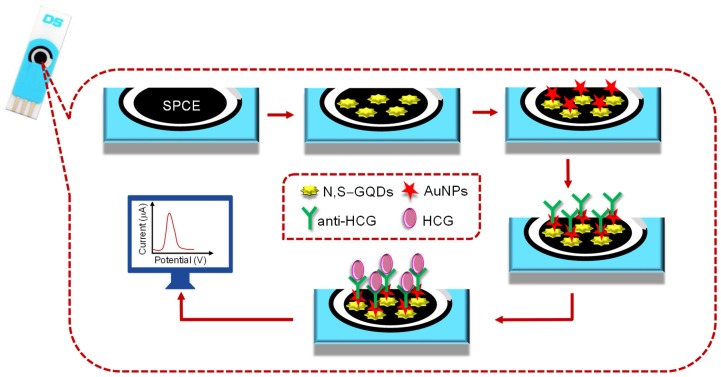
Various steps tangled in designing a GQD-based antibody sensor for tracking human chorionic gonadotropin (HCG) levels [79].

By taking riboflavin as a redox probe, Valipour et al. explored the functions of thiolated graphene quantum dots (GQD-SH) as well as AgNPs as electrode-enhancing agents for determining hepatitis C virus (HCV) in a spiked human serum [281]. AgNPs were loaded on GQDs–SH through the Ag-S bond and subsequently, anti-HCV antibodies were immobilized on the GCE. Using DPV, the specific immune-recognition was investigated through the decrease in oxidation signal response of the redox probe. This immunosensing platform displayed a broad linear range (0.05 pg mL^−1^ to 60 ng mL^−1^) with an LOD of 3 fg mL^−1^. Later on, in 2019, they fabricated a facile and reasonable voltammetric antibody-sensor to selectively and accurately determine human chorionic gonadotropin (HCG) [61]. AuNPs and N,S–GQDs were embedded stepwise onto the surface of SPCE (Figure 18). The as-functionalized SPCE enhanced the antibody capturing as well as amplified the signal for the sensitive analysis of HCG. To study the electrochemical characterization of SPCE, the electrode was then examined by ESI as well as CV methods. At the same time, DPV could determine HCG over 0.1–125 pg mL^−1^ concentrations, with an LOD of 12.5 fg mL^−1^. Furthermore, the reproducibility of the as-developed sensor was studied by carrying out the intra- and inter-assay precisions, which revealed that the nanocomposite functionalized SPCE retained good reproducibility as well as a broad potential window.

In 2018, Cai et al. reported an electrochemical sensing technique for calycosin, a biomarker responsible for inflammation, diabetic cognitive impairment and cardiac diseases [282]. In this work, polyaniline functionalized GQDs were produced by following an in situ polymerization. Their characterization was carried out by FT-IR and Raman spectroscopy, whereas the modified GCE was characterized by TEM and SEM. Owing to the CV and EIS results, the as-functionalized GCE could boost up the electron transferability. The DPV method revealed a broad linear regression function for calycosin, ranging from 11 to 352 μmol L^−1^, with a minimum detection capability of 9.8 μmol L^−1^.

**Figure 19:**
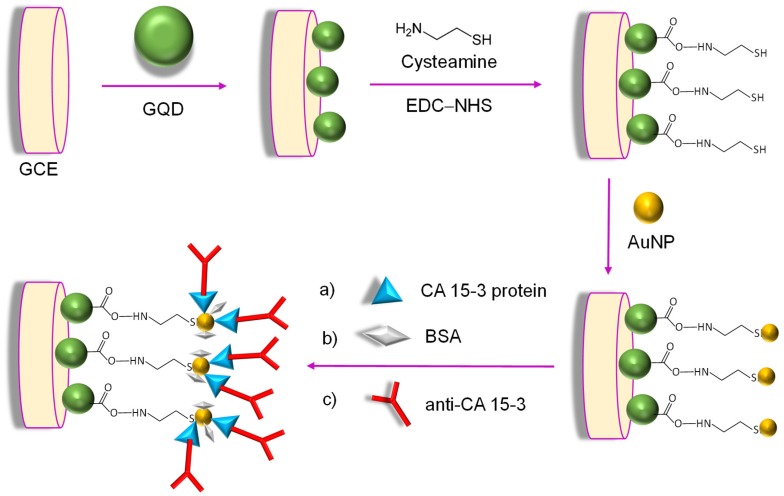
Experimental steps to fabricate a GCE for detecting breast cancer [79].

In 2018, Hasanzadeh and coworkers developed a ternary signal amplification approach for demonstrating a voltammetric antibody-sensor to sensitively detect the *p53* gene [283]. The experiment involved the immobilization of biotinylated p53 antibody onto a biocompatible and green nanocomposite layer comprising AuNPs/GQDs and poly L-cysteine (P-Cys) as amplifying components and polymeric matrix, respectively. An amalgamation of such nanomaterials enhanced the lateral surface of an Au-electrode for the effective immobilization of anti-p53 bio-receptors. Such a sensing system could exhibit a wide linearity of 0.0488–12.5 pM with an LOD of 23.4 fM. As shown in Figure 19, these researchers also designed an immunosensor for accurately recognizing CA 15-3, a tumor marker responsible for breast cancer [284].The electrode was functionalized by thiolated–GQDs, AuNPs and cysteamine (CysA). Consecutively, GQDs/AuNPs/CysA system offered abundant sites for capturing CA 15-3 molecules as well as the stability. The sensor could respond in a linear fashion of 0.15–125 U mL^−1^, with an LOD of 0.11 U mL^−1^.

A sandwich-type electrochemical sensor was designed for determining PSA in human blood sample [285]. The sensor construction process involved graphene sheets modified with GQDs for capturing anti-PSA molecules upon GCE. The electrochemical sensing of PSA could be achieved by SWV and offered a broad linear range from 0.005 to 10 ng mL^−1^ with an LOD of 3 pg mL^−1^, which was achieved because of the enhanced anti-PSA immobilization onto GCE as well as excellent electron transferability of GQDs.

In 2018, Mollarasouli and the group designed a voltammetric antibody-sensor to quantify a tyrosine kinase bio-receptor, i.e., AXL [286]. This protein is one of the major biomarkers responsible for the cardiac-arrest [287]. In this approach, 2-aminobenzyl amine (2-ABA) was used to modify GQDs@SPCE through electro-polymerization. To assure the adherence of GQDs onto the electrode surface, the functionalized SPCE was then incubated at 120 °C for about 60 min. Thereafter, the AXL antibodies were incubated on the electrode surface for 90 min. Using DPV, the target analyte was determined in a dynamic concentration range from 1.7 to 1000 pg mL^−1^ with the detection limit of 0.5 pg mL^−1^.

Tufa and colleagues proposed a sandwich-immunoassay sensor to quantify *Mycobacterium tuberculosis* antigen (i.e., CFP-10) using AuNPs and GQD-embedded Fe_3_O_4(core)_@Ag_(shell)_ nanocomplex as signal amplification labels and GCE enhancer, respectively [288]. This immunosensor exhibited an additive effect due to the multifarious role played by the nanomaterials residing within the nanocomplex, i.e., Ag accelerated the electron transferability, GQDs increased the surface area for the effective immobilization of anti-CFP-10 antibodies, and Fe_3_O_4_ enlarged the surface/volume ratio. The CFP-10 antigens were determined using DPV that offered a broad linear response from 5.0 ng mL^−1^ to 500 μg mL^−1^, with 0.33 ng mL^−1^ being the quantification limit.

An avian leukosis virus subgroup J (ALVs-J) was quantified by employing Fe_3_O_4_-fused GQDs and apoferritin-implanted Cu (apoferritin-Cu) NPs via a signal amplification strategy [289]. In the study, the researchers used GQDs to effectively immobilize anti-ALVs-J. On the other hand, apoferritin-Cu NPs were conjoined with the GQDs/Fe_3_O_4_ hybrid material. Subsequently, to release Cu from the apoferritin cavity, the immunochip was dipped in an acidic medium. The detection of ALVs-J antigen was accomplished by this biosensor in a linear range of 102–105 TCID_50_ mL^−1^, where an LOD of 115 TCID_50_ mL^−1^ was reported.

**Figure 20:**
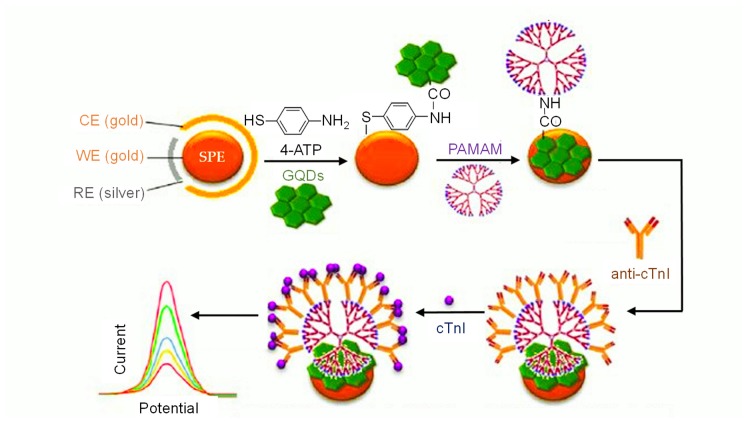
Schematic presentation of a voltammetric antibody sensor for cTnI [79].

Bhatnagar and the team engineered a voltammetric sensor for quantifying cTnI [290]. In this work, the electrode surface was initially laminated with 4-aminothiophenol (4-ATP), where the resultant NH_2_ groups were then covalently attached to the COOH groups of GQD through carbodiimide coupling chemistry. Moreover, the sensitivity of the sensor was improved by polyamidoamine (PAMAM) that was fused with GQD. Serving as the electrode enhancers, these nanomaterials could give a larger surface area to mount the antibodies. Subsequently, the activated cTnI monoclonal antibodies were immobilized on PAMAM dendrimer for probing cTnI antigens (Figure 20). The immunoreaction was examined with the help of DPV and CV, attaining an LOD of 20 fg mL^−1^ over a wide cTnI concentration range from 10^‒6^ to 10 ng mL^−1^. Very recently, Lakshmanakumar et al. designed a model for cTnI biomarker determination [291]. In this work, acetic acid functionalized GQDs (fGQDs) were used as an interface for cTnI, where the interaction between cTnI and fGQDs was investigated by CV as well as amperometry. The carbodiimide coupling reaction between the NH group of cTnI and the functionalized COOH group on GQDs was responsible for the identification of cTnI over a linear range of 0.17 to 3 ng mL^−1^ and an LOD of 0.02 ng mL^−1^. Besides, Table 4 features some critical aspects associated with several other recently reported GQD-sensors based on voltammetry.

### 3.2. Amperometric GQD Sensors

Amperometric sensors interpret the current flow generated at a constant voltage within the electrochemical cell. These sensors work on the principle of specific molecular bio-recognition to form a receptor-analyte complex. They can involve either a direct or indirect (i.e., non-labeled or labeled) assay approach. The former case determines physical changes caused during the formation of the receptor–analyte complex, while the latter case uses signal generating labels. It is worth noting that indirect mode is generally selected over the direct amperometry owing to its versatility and higher sensitivity [303]. These biosensors measure the potential applied between a RE and a WE to facilitate the redox reaction by calculating the resultant current [240]. Therefore, the current produced during an electrochemical reaction corresponds to the amount of signaling elements [304]. Figure 15D depicts the ideal plot of amperometric sensor signals.

Hu and co-workers designed a facile amperometric RNA sensor for quantifying miRNA by employing HRP-based catalysis [305]. This sensing system was fabricated by a ds-DNA. Herein, ds-DNA was hybridized on Au-electrode via the use of capture DNA probe (i.e., thiolated oligodeoxynucleotide), aminated indicator probe (NH_2_–DNA) as well as target DNA. Subsequently, the activated carboxyl groups of GQDs were functionalized on NH_2_–DNA via a non-covalent interaction. This HRP-modified sensor was able to catalyze the H_2_O_2_-mediated oxidation of TMB, guarded by a color change of a solution from transparent to blue and led to an amplified electrochemical current signal. Owing to the use of GQDs and enzyme catalysis, the as-prepared biosensor could sensitively quantify miRNA-155 in the range of 1 fM–100 pM, with an LOD of 0.14 fM, which could be ascribed to the good biocompatibility as well as greater surface/volume ratio offered by GQDs.

**Figure 21:**
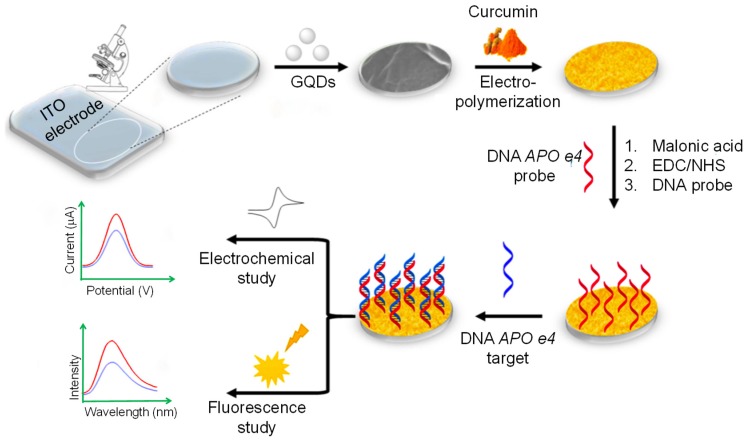
Set-up of an electrochemical and fluorescence based DNA-sensor for *APO e4* DNA [306].

As portrayed in Figure 21, Mars et al. introduced a DNA-sensor for targeting *APO e4* DNA in human blood, which causes Alzheimer’s disease [306]. Herein, electrochemical as well as fluorescence-sensitive curcumin and GQDs were electropolymerized on to an indium tin oxide (ITO)-coated electrode. An amino-functionalized DNA probe was then covalently implanted by EDC-NHS coupling chemistry to establish a complex consisting of hybridized DNA and curcumin. Amperometric measurements indicated that this strategy can be used to determine *APO e4* DNA in a linear calibration region of 20–400 × 10^−12^ g mL^−1^ and estimated an LOD of 0.48 × 10^−12^ g mL^−1^.

In 2017, a label-free electrochemical DNA-sensor for M.SssI activity determination was engineered, depending on the amplification of GQDs and an enzyme-catalyzed reaction [307]. The stepwise fabrication of this sensor involved the hybridization of a capture DNA with auxiliary DNA to develop a structure of ds-DNA, possessing a specific recognition sequence (5′-CCGG-3′) for both M.SssI and restriction endonuclease, HpaII. The ds-DNA was methylated by M.SssI, which was unable to be digested by HpaII, leading to the attachment of GQDs with this undigested ds-DNA that could immobilize HRP via a non-covalent assembly. Such a sensing platform could catalyze H_2_O_2_-mediated oxidation of TMP for an amplified electrochemical signal response. This DNA-sensor could monitor the M.SssI activity in a wide range between 1 to 40 U mL^−1^, with an LOD of 0.3 U mL^−1^. Moreover, this sensor was also applied to check an inhibitory effect of M.SssI for the screening and development of drugs like procaine and epicatechin.

In 2018, Dourandish and Beitollahi synthesized GQDs by altering the carbonization degree of citric acid, where they functionalized a graphite screen-printed electrode with these GQDs for an amperometric determination of isoproterenol [308]. In contrast to the unmodified electrode, the GQD-coated electrode caused an enhancement in signal response. The peak currents generated by the GQD electrode for isoproterenol were analyzed by CV, DPV as well as chronoamperometry, which exhibited a wide dynamic linear range from 1 to 900 μM, with an LOD value of 0.6 μM.

A sensitive non-enzymatic amperometric sensor was designed by Zhu et al. with long-term stability, good reproducibility and anti-interference. This sensor was fabricated using GQDs, AuNPs and polydopamine (PDA) as a catalyst, signal amplifying element and as an adhesive agent, respectively [309]. The synergistic effect of Au-PDA‒GQDs nanocomposite exhibited an excellent electro-catalytic activity towards the reduction of H_2_O_2_. This biosensor based on Au-PDA‒GQDs could quickly sense H_2_O_2_ (<2 s), with quite a broad linear relationship, ranging from 0.1 to 20,000 μM and an LOD of 5.8 nM.

Ju et al. developed an amperometric sensing strategy exploiting the use of N‒GQDs, where the surfactant-free AuNPs were grown on N‒GQDs in order to create abundant adsorption sites [310]. This hybrid nanocomposite was prepared by simply adding chloroauric acid (HAuCl_4_·4H_2_O) to N‒GQDs solution without mixing other surfactants and reductants. The resulting non-surfactant-capped AuNPs could deliver naked catalytic surface with strong electro-catalytic activity. This electrochemical sensor could detect hydrogen peroxide (H_2_O_2_) in human serum samples and H_2_O_2_ liberated from human cervical cancer cells with an LOD of 0.12 μM and a linear response in the range of 0.25−13327 μM.

Rezaei and Razmi used GQDs as an appropriate substrate for immobilizing an enzyme, GOx on a carbon ceramic electrode (CCE), where the quasi-reversible redox peaks were generated [311]. The UV–vis PL spectroscopy, TEM, SEM, EIS and CV methods confirmed the successful construction of the CCE, while amperometric measurements revealed the presence of glucose over the range of 5 to 1270 μM, with the quantification limit of 1.73 μM. An excellent performance of this biosensor can be ascribed to the greater surface-to-volume ratio, porosity of GQD@CCE, good biocompatibility of GQD and the numerous hydrophilic edges as well as the hydrophobic plane in GQD, which facilitated the enzyme immobilization on CCE. In 2016, the same group investigated the impact of electron transfer as well as the direct electrochemistry of hemoglobin on GQDs-chitosan nanocomposite (Hb‒GQDs-Chit) [312]. In this study, an amperometric sensor was constructed for detecting H_2_O_2_ in urine samples, which was characterized by TEM, PL, UV–Vis, XRD, CV and EIS techniques. The biosensor realized the sensitive detection of H_2_O_2_ in a broad spectrum from 1.5 to 195 μM, with an LOD of 0.68 μM. In the same year, Xi et al. also developed an amperometric biosensor for H_2_O_2_ for an early diagnosis of cancer, where they used carbon based hollow-structured nanospheres (HNSs) grafted with Pd NPs garnished N–GQDs as the dual signal-amplifying nanoprobes [313]. The fusion of these nanomaterials promoted the electron transfer as well as electro-catalytic activity for H_2_O_2_, due to the electrochemical reduction of H_2_O_2_ at 0 V, which could hinder the redox potential of several other electroactive agents in biological matrices by efficiently ignoring the interference signal response. The authors inferred that this biosensor could trace the minimum H_2_O_2_ concentrations of 20 nM, within just 2 s with high sensitivity, selectivity, stability and reproducibility.

**Figure 22:**
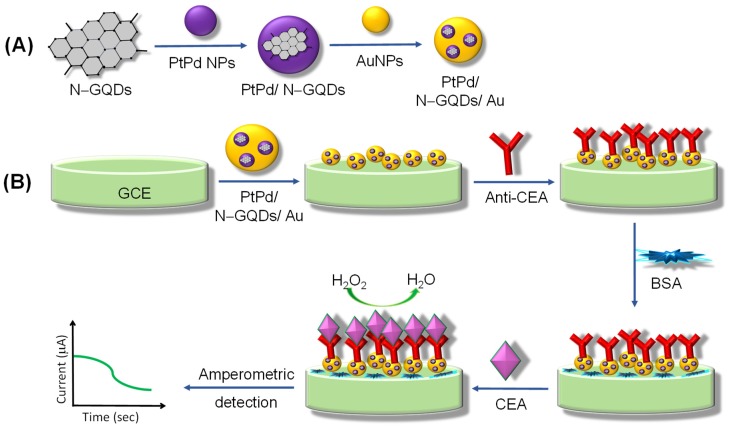
(**A**) Preparatory steps for PtPd/N‒GQDs/Au nanohybrid. (**B**) Schematic portrait of a label-free amperometric antibody-sensor for CEA determination. Adapted from Mansuriya and Altintas [79].

Yang and coworkers introduced a label-free sensing strategy, relying on antibody receptors [78]. This immunosensor was fabricated by modifying GCE with Au, PtPd and N‒GQDs for the selective identification of CEA. Using a self-assembly process, PtPd/N‒GQDs/Au hybrid was synthesized (Figure 22) through covalent interaction. The combined additive features provided by nanomaterials residing in the resultant hybrid material include larger surface area, enhanced electron transferability, excellent biocompatibility and ability to catalyze H_2_O_2_. Hence, these nanocomposites, acting as the signaling species, were employed for signal transduction as well as to effectively conjugate bioreceptors. PtPd/N‒GQDs/Au was used as a transducer to efficiently conjugate the capture antibodies and to act as a signal amplification system. The as-studied sensing system was highly specific for CEA because it could generate a negligible response for various interfering agents such as BSA, IgG, PSA and hepatitis B surface antigen (HBS). It was reported that this sensing tool could be capable of quantifying CEA down to 2 fg mL^−1^ with a linear regression function ranging from 5 fg mL^−1^ to 50 ng mL^−1^.

Muthurasu and Ganesh quantified H_2_O_2_ with the help of an enzyme-based amperometric sensor using GQDs that were prepared by modifying Hummer’s method [314]. In the study, HRP was conjoined with the GQDs via an acid–amine coupling to form a peptide-amide bond. The activity of HRP was determined through its drop-casting on GQDs-modified GCE, where the sensor could perform well for H_2_O_2_ from 100 μM to 1.3 mM, with an LOD of 531 nM. On the other side, Jiang et al. developed an enzyme-free approach for H_2_O_2_ sensing by functionalizing a gold electrode with GQDs, silver nanocubes and chitosan [315]. The Chit–GQDs/AgNCs electrode displayed an excellent electro-catalytic activity to reduce H_2_O_2_, revealing the amperometric measurements in a dynamic linear range of 10 μM to 7.8 mM, with an LOD of 0.15 μM.

Roushani and Abdi reported an electrochemical platform for sensing persulfate (S_2_O_8_^-2^) [316]. The sensor was developed by modifying GCE with GQDs and riboflavin, which was characterized by TEM, UV–vis and PL spectroscopy. The GQDs/riboflavin/GCE showed high stability and a wide array of pH (i.e., from 1 to 10) for redox couples having surface-confining features. Moreover, this GQD-sensor could readily catalyze the S_2_O_8_^−2^ reduction reaction and reflected an amperometric response of S_2_O_8_^−2^ over a wide linear range (i.e., from 1 µM to 1 mM), with an LOD of 0.2 µM.

**Figure 23:**
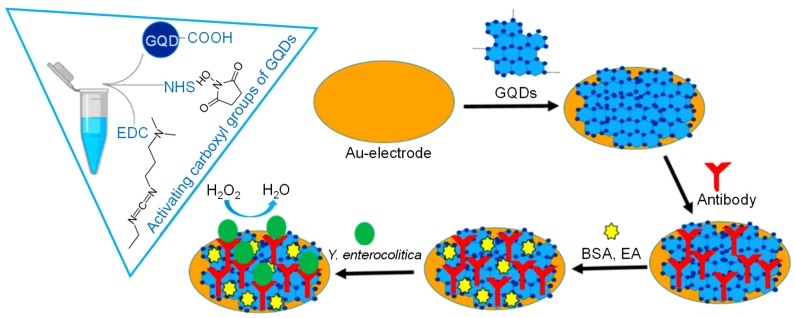
Schematic illustration of a GQD-based antibody sensor for *Y. enterecolitica* quantification [67].

In 2019, Altintas and Savas fabricated a novel label-free immunosensing platform for *Yersinia enterecolitica* (*Y. enterecolitica*) [67]. The sensor set-up was commenced through the functionalization of the Au electrode by GQDs, due to their excellent catalytic behavior to reduce H_2_O_2_. Herein, the specific antibodies were immobilized via the blockage of non-reacted COOH groups on an Au electrode, using both ethanolamine (EA) as well as BSA, as outlined in Figure 23. Thereafter, *Y. enterecolitica* was determined by amperometry, which depended on the inactivation of GQDs’ electro-conductivity due to the resultant immunoreaction. Correspondingly, elevated concentrations of *Y. enterecolitica* suppressed the amperometric signal response. This articulately formulated sensing device could demonstrate a broad linear relationship even in complex media such as human serum and milk in the range of 1‒6.23 × 10^8^ cfu mL^−1^, with an LOD of 30 cfu mL^−1^ and 5 cfu mL^−1^ in human serum and milk, respectively. Importantly, the matrix effect could not impact the sensing mechanism considerably, since the investigational array and the LOD values attained in a phosphate buffer saline were quite identical to that in biological samples (i.e., the sensor exhibited an LOD of 1 cfu mL^−1^ with amplified signal response in buffer as compared to the complex media). Moreover, the authors also stated that this antibody sensor is highly specific to *Y. enterecolitica*, as it showed negligible response against various other interfering bacteria such as *Yersinia pestis*, *E. coli*, *B. anthracis* and *Salmonella enteritidis*, suggesting the multifarious as well as an attractive tendency of GQD-immunosensors.

Various researches on interleukin-13 receptor α2 (IL13Rα2) have trotted out that its overexpression can form several cancerous cells including AIDS-associated Kaposi’s sarcoma, colorectal, glioma as well as squamous cancer of the neck and head [317]. In 2019, Serafín et al. demonstrated an amperometric antibody sensor to quantify IL-13Rα2 which entails the implantation of streptavidin-functionalized SPCEs with biotinylated-specific capture antibody via their treatment with p-amino benzoic acid (p-ABA) and further surface modification through carbodiimide-coupling chemistry [318]. A nanocomposite consisting GQDs and MWCNTs was chosen as an HRP mimic and a nanocarriers of the detector antibody, respectively. The involvement of such a nanocomposite enhanced the sensitivity remarkably owing to the GQDs’ peroxidase-mimicking catalytic performance. The electrochemical measurements for IL-13Rα2 through hydroquinone (HQ)/H_2_O_2_ assembly could show a quantification limit of 0.8 ng mL^−1^ over a broad linear concentration range (i.e., 2.7 to 100 ng mL^−1^). Such a sensing system can be clinically applied to selectively and rapidly detect IL-13Rα2 for the diagnosis of colorectal cancer. The same team has demonstrated a dual amperometric bio-assay for the concurrent determination of CDH-17 and IL-13Rα2, tumor markers responsible for colorectal and breast cancers, respectively [81]. Within this study, screen-printed dual carbon electrode (SPdCE) was assembled with GQDs/MWCNTs for establishing the sandwich immunoassay mode. Thereafter, the fabrication process of SPdCE as well as the detection technique were conducted in an identical manner as described in their aforementioned study. This immunoassay could provide the selective quantification of both tumor markers (i.e., CDH-17 and IL-13sRα2) with respective LOD values of 0.03 ng mL^−1^ and 1.4 ng mL^−1^.

**Figure 24:**
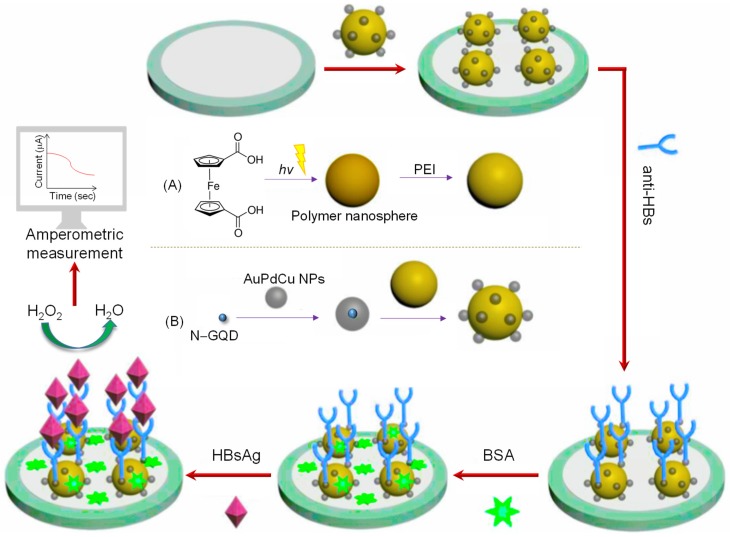
A picturized illustration of a label-free amperometric antibody-sensor for HBV virus and synthesis of: (**A**) polyethylenimine (PEI)-modified polymer nanospheres (PS); (**B**) N‒GQDs/AuPdCu @ PS. Adapted from Mansuriya and Altintas [79].

In 2018, a GCE-based label-free antibody sensor was proposed to determine hepatitis B surface antigen (HBsAg) [250]. As shown in Figure 24, the study aimed to implant surfactant-less AuPdCu ternary NPs onto N‒GQDs (N‒GQDs/AuPdCu), which offered excellent electro-conductivity and strong catalytic performance for H_2_O_2_ reduction. Additionally, polyethylenimine (PEI)-decorated electroactive polymer nanospheres (PS) was used as a transducer for delivering N‒GQDs/AuPdCu as well as for subsequently immobilizing the bioreceptors (i.e., anti-HBs antibodies). The stepwise fabrication of the electrode surface was characterized by EIS measurements, whereas amperometric detection of HBsAg revealed the linear relationship in the range of 10 fg mL^−1^–50 ng mL^−1^ and attained an LOD of 3 fg mL^−1^.

On the basis of a double-signal amplification approach, Malekzad and co-workers successfully quantified PSA, using mediator-free multilayer amperometric antibody sensor [319]. Herein, anti-PSA antibody was incubated with a biocompatible nanocomposite film of GQDS/AuNPs and poly L-cysteine (P-Cys) as dual-signal amplification elements and as a conductive matrix, respectively. This methodology was employed for developing a novel transduction platform on an Au surface that improved the surface of electrode for capturing abundant antibodies. Moreover, AuNPs were prepared by a soft template synthesis technique that attained a compact morphology. The as-functionalized electrode surface was then characterized by energy-dispersive spectroscopy (EDX) and high-resolution FE-SEM. The calibration curve for PSA concentration was linearly co-related in the range of 2–9 pg mL^−1^, with an LOD of 1.8 pg mL^−1^.

### 3.3. Impedimetric GQD Sensors

Numerous research works have been devoted to the conception of impedimetric or capacitive biosensors. EIS is acutely used to characterize the functionalized sensor surface, to examine kinetic interactions among various biological molecules like proteins, DNAs, receptors, antigens, antibodies, etc., and to establish label-free sensors [21,320]. The impedance measurements can be non-faradic or faradic; in other words, in the absence or presence of a redox probe. The latter measurements determine biochemical reactions occurring at the surface of a functionalized electrode through the interpretation of interfacial electron transfer resistance [320,321]. EIS converts an electrochemical signal response into a minute amplitude sinusoidal voltage response with respect to the frequency. As a result, current sine wave is generated, which changes with respect to the voltage wave and time (i.e., phase shift). This current to voltage ratio (*V*(*t*)/*I*(*t*)) generates an impedance (*Z*) [322,323]. For gaining the information of bio-recognition events taking place at the interface, a Randles equivalent circuit (i.e., simulated circuit) is generally employed for the determination of the mass-transfer resistance (R_mt_), the charge-transfer resistance (R_ct_), electrolyte resistance (R_el_), double-layer capacitance (C_dl_) and Warburg impedance (W) [324]. The Bode and Nyquist plots retrieve the EIS information [79]. In the Bode plot, frequency is plotted against the overall impedance *(|Z|*). In the case of the Nyquist plot, the real part of impedance (*Z’*) versus the imaginary part of impedance (*−Z’’*) is plotted (Figure 15E) [325]. The impedimetric sensors are efficient due to their easy instrumentation, lucrative electrode bulk manufacturing, miniaturization ability, and remote control of the grafted sensors [322].

**Figure 25:**
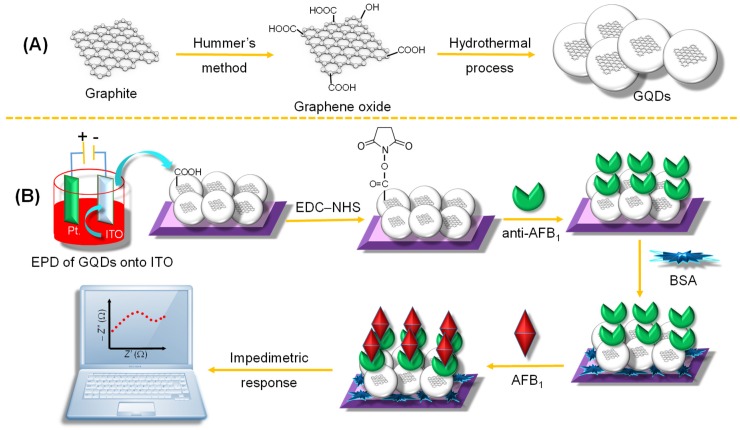
(**A**) Preparation of GQDs. (**B**) Immunosensing platform for AFB_1_ using GQD-functionalized indium tin oxide (ITO)-coated glass electrode [79].

Bhardwaj et al. reported a label-free impedimetric-sensing strategy using GQDs to detect aflatoxin B_1_ (AFB_1_), a food toxin [326]. GQDs were chemically prepared on the basis of Hummer’s method, as depicted in Figure 25 [327]. The sensor assembly was terminated by the electrophoretic deposition (EPD) of hydrothermally prepared GQDs on to an ITO-coupled glass electrode, following an immobilization of monoclonal AFB_1_-antibodies using EDC-NHS chemistry. The excellent electro-conductivity and increased surface area of the GQD sensor led to the detection of AFB_1_-contaminated maize samples in a broad linear range from 0.1 to 2.0 ng mL^−1^, with an LOD of 0.03 ng mL^−1^. Moreover, the researchers declared that this sensing platform could last its activity almost up to 7 weeks, indicating the long-term storage stability and better reproducibility.

A functionalized‒GQDs based impedimetric antibody-sensor was established by Mehta and the group for quantifying a pesticide, parathion [328]. For fabricating the sensor, GQDs were embedded on a SPCE surface, followed by its amine functionalization through the use of 2-ABA. Subsequently, the treated SPCE was immobilized with the bio-receptors. This EIS sensing platform achieved a linear array of signal response for parathion, ranging from 0.01 to 10^6^ ng L^−1^ with an LOD value of 46 pg L^−1^ in food and environmental specimens, even in the existence of paraoxon (i.e., the metabolite of parathion). Moreover, the authors claimed that the effective regeneration of this biosensor could be well established for five regeneration cycles using 10 mM glycine-HCl solution.

In 2018, Elshafey and Radi designed an impedimetric MIP-based sensor for the electrochemical determination of Alachlor (ALA), a carcinogenic herbicide found in groundwater, milk, eggs and meat [329]. The formation of MIP film onto GCE was undertaken through the electrodeposition of o-phenylenediamine (o-PD), where ALA was used as a template, followed by the removal of ALA to create specific recognition cavities for ALA. The stepwise construction of the sensor was characterized using CV, SWV and R_ct_, where the [Fe(CN)_6_]^3−/4−^ was employed as a redox probe. The controlled release pattern of the ALA from the MIP-o-PD network and the mole ratio of ALA to o-PD was optimized to create a maximum number of free imprinted sites, which influenced the sensitivity of the sensor. The increase in the R_ct_ value of the electro-active probe [Fe(CN)_6_]^3−/4−^ upon selective accumulation of ALA to the free voids of MIP film was determined as the sensor response, where the relative change in Rct was directly proportional to the ALA concentrations in a broad linear range from 1 nM to 1 μM, with an LOD value 0.78 nM ALA.

Employing an aptamer proximity-binding assay approach, Ghanbari et al. introduced an electrochemical aptasensor based on the functionalization of GCE surface by GQDs for the quantification of the hepatitis C virus (HCV) core antigen [330]. GQDs served as an appropriate substrate for aptamers via π–π stacking interactions to enrich the aptamer absorption on the GCE surface. Surface functionalization at each stage was characterized by EIS, CV and DPV, where the EIS technique was also used for efficiently determining HCV core antigen in human serum samples, which exhibited the detection limit of 3.3 pg mL^−1^ and a wide linear relationship ranging from 1 to 400 pg mL^−1^ HCV concentration. Later, in 2018, the same group had proposed another aptasensor based on thiolated GQDs (GQDS–SH) and AuNPs for sensing streptomycin (STR) [331]. In this study, a GQD–SH was integrated onto the surface of a GCE, followed by the decoration of AuNPs on to the thiol groups of GQDs via the formation of an Au–S bond. At the same time, aptamers were injected onto the GCE surface by interacting with the thiol groups of an aptamer and then a sample containing STR was incubated, resulting in an aptamer/STR complex formation. The proposed nanoaptasensor could detect STR by EIS in a broad linear range from 0.1 to 700 pg mL^−1^, with an LOD of 0.033 pg mL^−1^.

**Figure 26:**
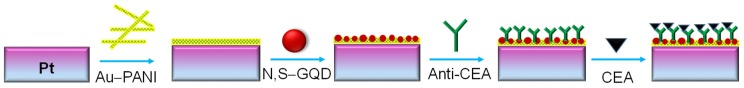
A label-free impedimetric sensing system for CEA. Adapted from Mansuriya and Altintas [79].

In 2019, Ganganboina and co-workers fabricated a label-free antibody-sensor using N,S–GQDs and gold-embedded polyaniline (Au-PANI) nanowires for the sensitive and selective identification of CEA [332]. N,S–GQDs served as the bifunctional probe to immobilize anti-CEA and to improve the electrochemical performance. Figure 26 indicates the deposition of Au-PANI onto the Pt electrode, followed by the lamination of N,S–GQDs onto the Au-PANI surface through the Au-thiol bond. The change in impedance of Au-PANI/N,S–GQDs in the presence of CEA suppressed the electron transfer due to the immunoreaction between CEA and its specific antibody on the surface of Au-PANI/N,S–GQDs. This label-free antibody sensor provided a broad linear range of CEA concentration from 0.5 to 1000 ng mL^−1^ with an LOD of 0.01 ng mL^−1^. In 2019, another impedimetric immunosensor exploring the use of these nanomaterials, i.e., N,S–GQDs and Au-PANI nanowires, was constructed by Chowdhury and colleagues for quantifying hepatitis E virus (HEV) [333]. Herein, the WE was implanted with Au-PANI nanowires and N,S–GQDs by a self-assembly and an interfacial polymerization strategy. The Au-PANI/N,S–GQDs nanocomposite was covalently immobilized by anti-HEV and an external electrical pulse was induced during the injection of the HEV antigen to boost sensitivity towards the target virus due to the surface expansion of the virus as well as to increase the length of the anti-HEV-assembled polyaniline chain. This EIS biosensor could efficiently determine and distinguish various HEV genotypes from human serum and from fecal samples of a HEV-infected monkey, with the LOD values of 96.7 RNA copies mL^−1^ and 0.8 fg mL^−1^, respectively. Surprisingly, this sensor could display identical sensitivity to that exhibited by a real-time quantitative reverse transcription polymerase chain reaction (RT-qPCR).

**Figure 27:**
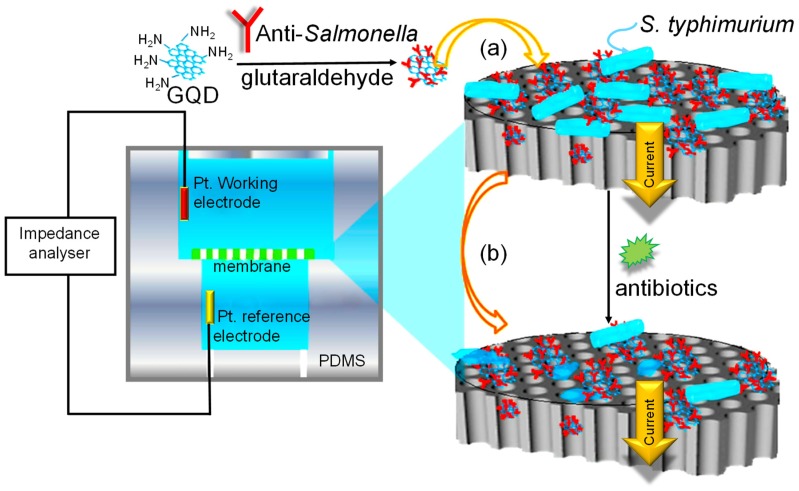
Detection of *S. typhimurium* by an impedimetric antibody sensor, followed by its response towards antibiotics, where (**a**) increased impedance (**b**) decreased impedance [79].

Ye et al. demonstrated an impedimetric immunosensor for the sensitive detection of *Salmonella typhimurium* [334]. Figure 27 portrays a biosensor set-up that comprises of a nanoporous alumina membrane and a polydimethylsiloxane (PDMS) chamber, where the detection of *S. typhimurium* was influenced by the variation in impedance through the membrane. When the membrane was clogged by the accumulation of *S. typhimurium*, impedance increased, followed by the decrease in impedance and death of the bacteria due to the introduction of antibiotics. Hence, this capture/sensing strategy was examined to quantify the target bacteria as well as to examine the response of target bacteria toward antibiotics. In the sensor development, modified GQDs were used to enhance the efficiency of the membrane by increasing its surface/volume ratio. Subsequently, anti-*Salmonella* antibodies were immobilized onto the membrane enriched with GQDs using glutaraldehyde as a cross-linking agent, which could detect *S. typhimurium* within just 10 min, and provided an LOD of 1 pM. Moreover, it was proved that this EIS sensor was extremely specific for *S. typhimurium* with a negligible response to other bacteria, suggesting its convincing clinical application even in the case of various other bacterial infections.

Tuteja et al. developed a label-free impedimetric immunosensor for sensing cardiac myoglobin (cMyo) [77], a major biomarker for the detection of MI [335]. In this work, GQDs were hydrothermally synthesized and implanted on screen-printed electrodes (SPEs) as an immobilized template. Subsequently, SPEs were incubated with the anti-cMyo antibodies to sensitively detect the cMyo. The modified SPEs displayed a wide range of 0.01–100 ng mL^−1^ and the detection limit was observed to be 0.01 ng mL^−1^ (i.e., >400 fold in comparison to the conventional enzyme-linked immunosorbent assay (ELISA) tests).

Beta-blockers (BB) are generally used as prophylactic drugs for atrial fibrillation (AF), which is common during open heart surgery [336]. Despite a number of studies during the last few years, satisfactory prevention of AF in individuals suffering from post-coronary artery bypass graft surgery (CABG) has not flourished through the use of these BB. The relation between AF and elevated C-reactive protein (CRP) levels is still contentious, if high plasma CRP concentrations are responsible for the cause of AF, or if the elevated CRP concentration is the reason behind the existence of this disorder [337,338]. Bing and team exploited the role of GQDs as a GCE enhancer to fabricate an efficient impedimetric antibody sensor for determining CRP in human plasma, particularly in case of clinical AF after CABG [339]. The stepwise fabrication of the GCE surface was characterized by the EIS technique and the target-specific R_ct_ values displayed a wide linear logarithmic CRP concentration in the range of 0.5–70 nM, with an LOD of 176 pM.

## 4. Photoelectrochemical and Miscellaneous GQD Sensors in Biomedical Diagnostics

According to our statistical analysis, only a few studies have recently reported on photoelectrochemical (PEC) GQD sensors in comparison to optical and electrochemical sensors. At the same time, various studies have been carried out using SPR sensors for drug monitoring and disease detection [340,341,342,343], but surprisingly only two such studies have reported on the use of GQDs. Given this, despite SPR sensors being optical sensors, we have compiled them in this section. Also, the biosensors like lab on chip [344], microfluidic [345], colorimetric, piezoelectric or quartz crystal microbalance (QCM) [346] and resonance light scattering (RLS)-based sensors are still under-developed. In spite of the versatility of piezoelectric sensors, no GQDs-based QCM sensor has been reported so far. Among these sensors, those working under the principle of PEC are still comparatively well studied. Therefore, we initiate the discussion of this section with PEC sensors, followed by the other sensors.

The PEC biosensing strategy originated from electrochemical techniques, which mainly involves the photoelectric conversion process as well as the influence of light on interface materials or photoelectrodes [347,348]. Photoelectrochemistry seems to be an interesting research hotspot because of its prominent attention from scientists, owing to its cost-effectiveness, rapid analytical response, easy miniaturization, simple instrumentation, exhibition of significantly low background signals and desirable sensitivity [349,350,351,352]. The PEC biosensors work on the principle of photocurrent signal that is generated during the binding event taking place between the target analyte and bioreceptor, which is associated with the energy and charge transfer of the PEC reaction upon light irradiation between the photoactive element and electron donor/acceptor moieties [351]. The photocurrent generation mechanism primarily depends on the photoexcitation of photoactive materials, which subsequently transfers electrons to the conduction band from the valence band. In turn, the desired pairing of an electron-hole pair resulted, followed by the elimination of conduction-band electrons and an anodic photocurrent signal is produced [349]. Nonetheless, the research on its detection mechanism still lies at ground level and thus, it needs to be further explored.

**Figure 28:**
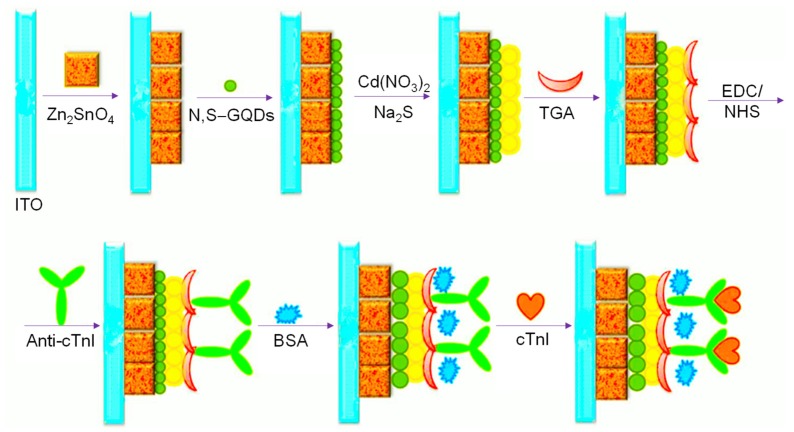
Fabrication of an N,S‒GQD-based photoelectrochemical (PEC) antibody-sensor for cTnI monitoring [353].

In 2018, a PEC immunosensor for determining cTnI levels was engineered using CdS and N,S‒GQDs co-sensitized solvothermally strategized cube of Zn_2_SnO_4_ (Figure 28) [353]. This Zn_2_SnO_4_ cube could effectively enhance the ITO electrode’s surface for the further modifications. N,S‒GQDs were then implanted to the Zn_2_SnO_4_ cube that boosted the electronic transferability as well as photo-to-current convertibility. Thereafter, CdS nanoparticles were functionalized to obtain CdS/N,S‒GQDs/Zn_2_SnO_4_ film. During the specific recognition of cTnI, the immunoreaction between cTnI and anti-cTnI decreased the photoelectric signal intensity from 0.001 to 50 × 10^−9^ g mL^−1^ of cTnI concentrations, which revealed an LOD of 0.3 × 10^−12^ g mL^−1^. Furthermore, the authors claimed that this PEC sensor could exhibit successful outcomes in MI diagnosis.

Another PEC immunosensor was reported by Tian et al. using GQDs conjugated with the oriental construction of silicon nanowires (SiNWs) to test microcystin-Leucine-Arginine (MC-LR), a toxic heptapeptide found in H_2_O_2_ samples [354]. A biocompatible nanoscaffold was engaged to immobilize the antibody as well as a SiNWs/GQD hetero-structure for signal transduction. The results obtained showed that the SiNWs/GQD system could perform well for specifically recognizing MC-LR, ranging from 0.1 to 10 µg L^−1^, with an LOD of 0.055 µg L^−1^, where the presence of MC-LR influenced the optoelectronic features of the as-functionalized surface of electrode. Hence, this sensor led to suppressed photocurrent signal intensity.

In 2018, Ahmed and co-workers demonstrated an optoelectronic immunosensing platform using gold nanobundles (AuNBs) and GQDs for detecting fowl adenoviruses (FAdVs) [355]. This strategy employed a sequential layer-by-layer (LbL) process for casting an AuNBs layer on carbon electrode system through the use of gold chloroauric acid, poly-l-lysine (PLL) and L(+) ascorbic acid. The nanohybrid comprised of GQDs and AuNBs coupled with anti-FAdVs receptors prior to quantifying the target virus. On injecting the FAdVs antigens, an amplified signal provided upto 10 pfu mL^−1^ of the target analyte with an LOD of about 8.8 pfu mL^−1^. This immunoassay could yield excellent sensitivity (i.e., more than 100 times) as compared to the standard ELISA tests.

In 2017, Ge at al. designed a PEC biosensor for expressing N-glycan, depending on the properties offered by nanogold-conjoined mesoporous silicon NPs (GMSNs) fused with localized SPR and GQDs [356]. In the development of a desired sensor, porous zinc oxide (ZnO) spheres were integrated on the Au nanorod-functionalized paper WE and were modified with the assistance of CdTe QDs. Subsequently, the immobilization of GMSNs onto CdTe QDs was conducted. Moreover, with the help of localized SPR, CdTe QDs were activated and the photocurrent efficacy was amplified. Thereafter, the multibranched hybridization chain reaction (mHCR) products to bind with HRP enzyme and to capture the target biomolecules were implanted on the surface of the WE, which resulted in exciting photoactive substances for facile instrumentation via a CL emission. Moreover, an aptamer could specifically capture the cancerous cells by its ability to recognize cells that ignored the conventional routing cell counting steps. At the same time, the GQDs acted as the signal amplification elements, exerting the process of N-glycan evaluation, because the H_2_O_2_ consumption and the competitive absorption of exciting light oxidized the luminol and the electron donor of the PEC system, respectively. This highly sensitive biosensing platform for monitoring the N-glycan-based physiological processes could attain an LOD of 21 cells mL^−1^, which can be used to examine glycomics in clinical diagnostics.

In 2019, Prado and the team constructed a PEC sensor for dopamine (DA) by exploring the activity of GQDs assembled on a fluorine-functionalized tin oxide (FTO) glassy electrode upon bio-immobilization of bismuth vanadate (BiVO_4_) [357]. Since GQDs can enhance the photocatalytic activity of BiVO_4_ by eliminating the photo-induced electrons from the conduction band through quantum confinement, the photocurrent signals generated were found to be in direct correlation with the DA concentration from 36 nmol L^−1^ to 250 μmol L^−1^, with a quantification limit of 2.7 × 10^−8^ mol L^−1^ [21]. Another GQD-sensor for DA detection was proposed by Yan et al., where GQDs were decorated with TiO_2_ nanoparticles through physical adsorption [358]. Under visible-light irradiation, these nanocomposites revealed enhanced PEC signal amplification, i.e., it increased almost 12 times and 30 times when compared to GCE/TiO_2_ and GCE/GQDs, respectively. This can be ascribed to the combined additive effect of GQDs and TiO_2_. Moreover, the photocurrent of these nanocomposites was elevated with the increase in DA concentrations from 0.02 to 105 µM and offered an LOD of 6.7 nM.

**Figure 29:**
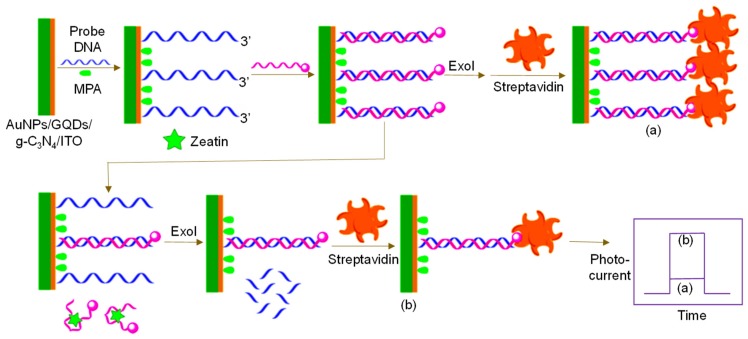
Design of a PEC aptasensor for zeatin determination using GQDs, AuNPs, g-C_3_N_4_ and DNA biotin-labeled aptamer [359].

In 2018, a PEC technique was established for quantifying zeatin, a cytokinin governing plant cell differentiation and proliferation [359]. As depicted in Figure 29, this method was coupled with streptavidin, graphite-like carbon nitride (g-C_3_N_4_), GQDs, AuNPs as well as a DNA biotin-labeled aptamer as PEC inhibition factor, photoactive reagent, photo-activity enhancing element, immobilization substrate for the DNA probe as well as zeatin recognition and streptavidin capture material, respectively. In this work, the DNA aptamer could bind to zeatin for the complex formation to liberate the aptamer from the electrode and decreased the capture amount of streptavidin, producing an enhanced PEC signal intensity. This DNA aptasensor could show a broad linear response from 0.1 to 100 nM zeatin concentration and attained an LOD value of 0.031 nM. Additionally, this technique could easily discriminate zeatin analogues in biological samples, suggesting its high selectivity.

Another PEC aptasensor for chloramphenicol (CAP) identification was introduced by employing a label-free aptamer and N‒GQDs as bio-recognition and transducer elements, respectively [360]. Hydrothermally synthesized N‒GQDs were exploited to generate an amplified photon-to-electron convertibility under visible-light irradiation. Moreover, the π-conjoined rigidity of N‒GQDs could exhibit an improvised surface to immobilize an aptamer through π-π bond. This N‒GQDs-aptasensor responded linearly from 10 to 250 nM of the CAP amount with a minimum quantification limit of 3.1 nM.

**Figure 30:**
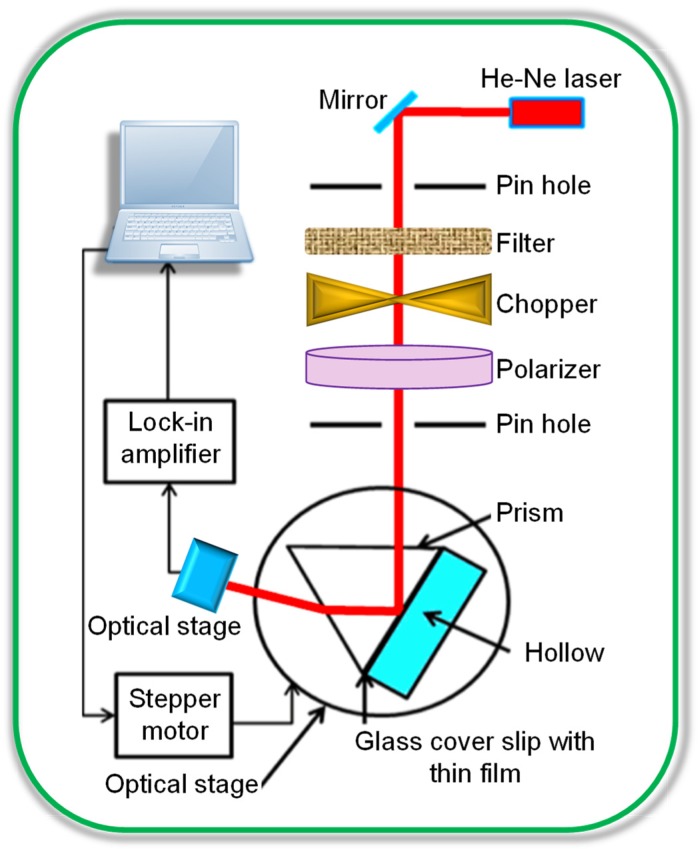
Schematic representation of an surface plasmon resonance (SPR)sensor. Adapted from Ramdzan et al. [361].

Recently, Ramdzan et al. designed an SPR sensor using chitosan and carboxyl-functionalized GQDs [361]. The existence of C=O, C–O, C–H and O–H bonds in a thin film comprising GQDs and chitosan was confirmed by FT-IR spectra, while its optical characteristics were approved by UV–Vis and PL spectroscopy. The PL spectra not only revealed the high absorbance value of chitosan/GQDs film with an optical band gap of 4.012 eV, but also gave an idea about the emission of red light because of the absorption peak appearing between 725 to 775 nm, which corresponded to π→π* transitions of C=O bond. This GQD-based SPR sensor was employed for the detection of Hg^2+^ contaminants, which could respond to Hg^2+^ in a linear fashion between 0 to 100 ppm, with an LOD value of 0.5 ppm. Figure 30 outlines the general instrumentation that works on the SPR principle. In 2019, Sadrolhosseini et al. developed a biosensor based on the principle of SPR, where polypyrrole-chitosan/GQDs was electrodeposited on a gold-coated glass chip [362]. The as-formed film was characterized by FE-SEM as well as XRD and the sensor could quantify glucose, fructose as well as sucrose. It was found that the sensor exhibited higher sensitivity for glucose than for fructose and sucrose. The authors reported that the detection limit of about 1 ppm was estimated for these sugars and the device could sense them within just 5 min.

**Figure 31:**
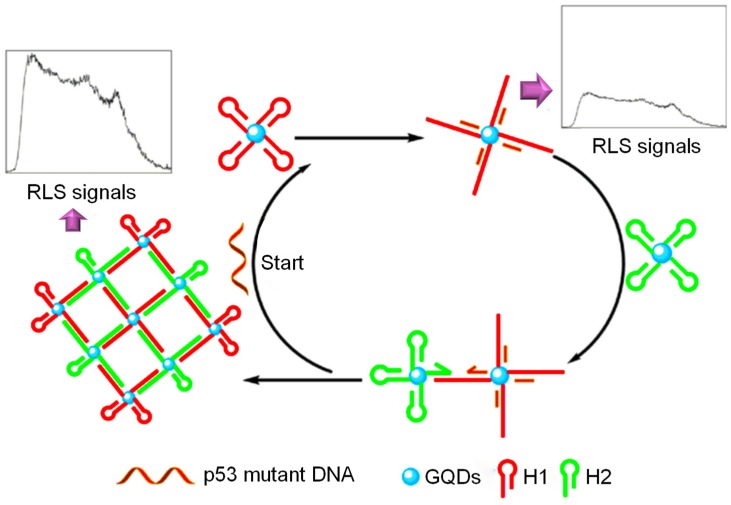
The mechanism of RLS of GQDs for detecting mutant DNA based on catalytic hairpin assembly amplification approach [363].

Using the RLS technique, an enzyme-free GQD-sensor was constructed in 2019 [363]. In this study, a catalytic hairpin assembly amplification approach was used for detecting p53 mutant DNA. Figure 31 pictures the development of this strategy with two probes comprising GQDs and hairpins in each (i.e., GQDs-H1 and GQDs-H2). Two partially complementary hairpins (i.e., H1 and H2) were so aptly framed that they could not undergo concurrent hybridization, when the target DNA is not present. As soon as the target DNA was introduced, it could bind with GQDs-H1 followed by the formation of GQDs-H1: target intermediates. Subsequently, GQDs-H2 could fuse with GQDs-H1, resulting in another reaction cycle. The generation of an increased RLS signal intensity could be ascribed to the aggregation of GQDs-H1 and GQDs-H2. This RLS-sensor could identify p53 mutant DNA in a broad range from 1 pmol L^−1^ to 50.0 nmol L^−1^, with the detection limit of 0.8 pmol L^−1^. Another RLS-sensor was reported by Wu et al. in 2017 [364]. The GQDs were selected as probes to fabricate this sensor for tracing proteins. According to the electrostatic interaction taking place between proteins and GQDs, the RLS signal was elevated, because of the incorporation of proteins in the GQDs solution. This RLS-sensor could provide a linear response, ranging from 10 μM to 60 μM protein concentration and an LOD of 2.4 μM.

Liu and team introduced a microfluidic paper-based approach for CA 15-3 biomarker quantification [365]. Au nanocages were deposited on the surface of a paper working electrode (PWE) for loading primary anti-CA 15-3 antibodies. Using the ECL technique and by immobilizing labeled secondary antibodies to establish a sandwich assay, the detection of a target analyte was accomplished. The characterization of the sensor surface was done using SEM, which revealed that the filamentous morphology of Au nanocages allowed more GQDs to be accumulated, permitting an increased ECL intensity. The as-developed sensor could offer a dynamic linearity over the range of 0.005–500 U mL^−1^ CA 15-3, with a quantification limit of 0.0014 U mL^−1^. Moreover, Li et al. also proposed a microfluidic paper-based sensing tool, where they explored the role of GQDs, concanavalin A and Au@Pd alloy NPs to set-up an assembly by layer-by-layer modification approach [366]. This device could efficiently sense H_2_O_2_ that oozed out from cancer cells on the basis of an in situ hydroxyl radicals cleaving DNA strategy, achieving an LOD down to 0.05 nmol.

**Figure 32:**
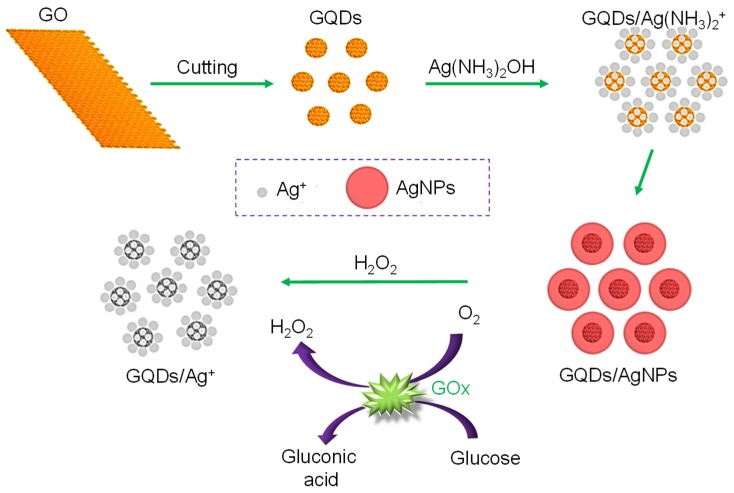
Preparation of GQDs/AgNPs nanocomposite for colorimetric detection of H_2_O_2_ and glucose. Adapted from Chen et al. [369].

To the best of our knowledge, the very first multifunctional GQD sensor based on the principle of colorimetry was invented by Park et al. in 2015 [367]. This biosensor involved the use of block copolymer-implanted GQDs (bcp‒GQDs), which generated variation in luminescence with respect to the change in pH and temperature that delivered the dose-dependent relationship corresponding to the distinct metal ions. This sensing platform could detect Fe^3+^ concentrations in a linear range of 0‒50 μM. In the same year, Lin and coworkers developed an N‒GQDs-based colorimetric sensor for monitoring H_2_O_2_ and glucose levels [368]. The linear regression function could lie over the range of 20‒1170 μM for H_2_O_2_ and 25‒375 μM for glucose, with LOD values of 5.3 μM and 16 μM for H_2_O_2_ and glucose, respectively. The authors reported that this method can be used to check the glucose contents in human serum as well as in fruit juice. As illustrated in Figure 32, another colorimetric sensor was proposed for H_2_O_2_ determination, where AgNPs and GQDs were employed for its development [369]. The GQDs/AgNPs nanocomposite showed an excellent absorbance-fading response for H_2_O_2_ reduction. This colorimetric sensing platform was further applied for glucose detection based on the catalytic activity of glucose oxidase to oxidize and form glucose and H_2_O_2_, respectively. Such a sensing system could detect H_2_O_2_ as well glucose in the respective ranges of 0.1−100 μM and 0.5−400 μM, where the corresponding LOD values were reported as 33 nM and 170 nM. On the other hand, Nirala et al. presented a GQD-based colorimetric sensor for cholesterol detection [370]. Owing to the electro-catalytic activity offered by the GQDs in the presence of H_2_O_2_, a color change of a solution containing TMB was observed from transparent to blue. This sensing mechanism could deliver the quantification of cholesterol over a wide range from 0.02 to 0.6 mM, with an LOD of 0.006 mM.

## 5. Summary, Critical Issues and Future Directions

Nanomaterials like GQDs, alone or when conjoined with numerous other nanomaterials to form hybrid nanocomposites, exhibit marvelous features by virtue of their unique characteristics and/or their desired synergism for fabricating biosensors. Over the past decade, these biosensing platforms offered a plethora of opportunities to diagnose a wide range of diseases from autoimmune, neurodegenerative, cardiovascular and infectious diseases to cancer diagnosis. In association with the degree of particularity in the case of these pathophysiologies, their early detection via the use of disease biomarkers, toxins and pathogens in biological, environmental or/and food samples illustrates significant and concrete ideas about their severity. The sensitivity, selectivity, accuracy and reliability of the disease-associated molecules seem to be still challenging because of their ultralow concentrations. Nonetheless, it is evinced from our review article that the GQD-based sensors have now conspicuously accomplished the minimum limit of sensing certain target biomolecules. These biosensors for early disease detection have gained meticulous popularity in many sectors such as clinical therapy, disease monitoring, discovery of preventive therapies as well as in the development of treatment-based medicines.

In this review, we examined research works conducted especially in last 5 years, highlighting the fascinating contribution of GQDs in introducing biosensors owing to their extraordinary and multifarious properties, i.e., good electro-catalytic activity, excellent fluorescence attributes, abundant modification sites, enhanced electro-conductivity, ability to amplify sensor signals, edge-effects, size effects, photo-stability, etc. Therefore, GQDs stand in such a position that they can easily functionalize several bio-electrodes, permitting quick, simple, stable, reproducible as well as lucrative sensing systems for clinical and practical applications. Moreover, these GQD-sensors are quite renowned for their excellent specificity, superior selectivity, and sensitivity in biological matrices including human blood, urine, sputum, saliva, milk, hard water, soil, etc., which can be ascribed to the physical and chemical attributes of GQDs.

Contradictorily, the involvement of costlier nanomaterials in combination with GQDs, laborious assay methodologies, insufficient storage stability and a number of displeasing factors at the nanoscale level are some obstructions that act as hurdles for their bulk manufacture. Additionally, most of these sensors reported very recently are not yet validated for their clinical applicability. Hence, strategies for establishing bulk batches of sensors and achieving their mass production as well as their approval in the real world have to be developed.

As far as our detailed and comprehensive literature survey is concerned, more than 80% of GQD-based sensors for biomedical applications have been reported from 2015 onwards, which suggests that GQD sensors represent one of the hottest emerging research topics. In terms of the overall statistics regarding the studies associated with GQD biosensors, to the best of our knowledge nearly 52%, 40% and 4% of the research works have been carried out to develop optical, electrochemical and photoelectrochemical biosensors, respectively. This can be credited especially to the mesmerizing fluorescent and electro-catalytic characteristics of GQDs. Surprisingly, only 4% of research works are based on other GQD sensors in total (i.e., piezoelectric, RLS-dependent, microfluidic paper-based and colorimetric sensors). Presumably, the substantial role of GQDs to construct biosensors for the clinical diagnosis of certain diseases will be developed in the near future.

## Figures and Tables

**Table 1:** Metal ion detection using fluorescence based GQD sensors for biomedical applications.

Nanomaterials	Target Metal Ion	Linear Range	LOD	Reference
GQDs	Cu^2+^	0–15 µM	0.226 µM	[116]
r–GQDs/GO	Pb^2+^	1–400 nM	0.6 nM	[117]
GQDs@AuNPs	Pb^2+^	50 nM–4 µM	16.7 nM	[118]
N,S–GQDs	Hg^2+^	0.1–15 µM	0.14 nM	[119]
Val–GQDs	Hg^2+^	0.8 nM–1 µM	0.4 nM	[120]
S–GQDs	Ag^+^	0.1–130 µM	30 nM	[121]
GQDs@OPD	Ag^+^	0–115.2 µM	250 nM	[122]
GQDs@AgNPs	Ag^+^	0–100 nM	3.5 nM	[123]
Cit-UCNPs/GQDs	Ag^+^	2 × 10^−4^–1 µM	60 pM	[124]
N–GQDs	Fe^3+^	1–1945 µM	90 nM	[125]
RBD–GQDs	Fe^3+^	0–1 µM	0.02 nM	[126]
N–GQDs	Fe^3+^	1–500 µM	1 µM	[127]
B–GQDs	Fe^3+^	50 nM–420 µM	31.2 nM	[128]
DA–GQDs	Fe^3+^	20 nM–2 µM	7.6 nM	[129]
S–GQDs	Fe^3+^	0.01–0.70 µM	4.2 nM	[130]

Abbreviations: Ag^+^: Silver ion; B–GQDs: Boron doped GQDs; Cit-UCNPs: Sodium citrate functionalized up conversion nanoparticles; Cu^2+^: Copper (II) ion; DA–GQDs: Dopamine-functionalized GQDs; Fe^3+^: Ferric (III) ion; GO: Graphene oxide; GQDs: Graphene quantum dots; Hg^2+^: Mercury (II) ion; N–GQDs: Nitrogen-doped GQDs; N,S–GQDs: Nitrogen and sulfur-doped GQDs; OPD: o-phenylene diamine; Pb^2+^: Lead (II) ion; RBD–GQDs: Rhodamine-functionalized GQDs; r–GQDs: reduced GQDs; N–GQDs: Nitrogen-doped GQDs; Pb^2+^: Lead (II) ion; Val–GQDs: Valine-functionalized GQDs.

**Table 2:** Various GQD sensors based on fluorescence for biomedical applications developed over the last five years.

Nanomaterials	Receptor Type	Receptor	Target Analyte	Linear Range	Detection Limit	Sample(s)	Reference
GQDs	Nucleic acid	G-quadruplex/hemin DNAzyme	Uric acid	2–300 µM	500 nM	Human serum; Urine	[143]
N–GQDs	MIP	PDA-imprinted polymer	Thiacloprid	0.1–10 mg L^−1^	0.3 mg L^−1^	Vegetables	[144]
GQDs	Enzyme	HRP	o-DHB; m-DHB; p-DHB	0.5–250 nM; 1–120 nM; 0.5–90 nM	2 × 10^−10^ mol L^−1^; 8 × 10^−10^ mol L^−1^; 3 × 10^−10^ mol L^−1^	Rain water; tap water	[145]
GQDs/MoS_2_ nanosheets	Nucleic acid	Aptamer	EpCAM	3–54 nM	450 pM	Human serum	[146]
GQDs	MIP	Atropine-imprinted polymer	Atropine	0.5–300 × 10^−9^ g mL^−1^	0.22 × 10^−9^ g mL^−1^	Urine; Human blood	[147]
GQDs/Fe_3_O_4_	Antibody	Anti-IgG	IgG	2.0–2.0 × 10^3^ casts mL^−1^	2.0 casts mL^−1^	Urine	[148]
N–GQDs	Nucleic acid	ss-DNA	Bleomycin	0.34–1300 nmol L^−1^	0.34 nmol L^−1^	Human serum	[149]
GQDs	Enzyme	Urease	Urea	0.1–100 mM	0.01 mM	Human blood	[150]
GQDs	MIP	tetracycline-imprinted polymer	tetracycline	1–10^4^ µg L^−1^	1 µg L^−1^	Milk	[151]
NH_2_–GQDs	Nucleic acid	G-quadruplex/hemin DNAzyme	Human telomere DNA	0.2–50 pM	25 fM	Buffer; Human blood	[152]
Silica–GQDs	MIP	4-NP imprinted polymer	4-NP	0.02–3 µg mL^−1^	9 ng mL^−1^	water	[153]
GQDs/p-ABA	Enzyme	Tyrosinase	Artemisinin	0.1–55 µM	33 nM	Pharmaceuticals	[154]
GQDs	MIP	Silica-MIP	Ornidazole	0.75–30 µM	0.24 µM	Human plasma	[155]

Abbreviations: 4-NP: Para-nitrophenol; DNA: Deoxyribonucleic acid; DHB: Dihydroxybenzene; EpCAM: Epithelial cell adhesion molecule; Fe_3_O_4_: Ferric ferrous oxide; GQDs: Graphene quantum dots; HRP: Horse-radish peroxidase; IgG: Immunoglobin-G; MIP: Molecularly imprinted polymer; MoS_2_: Molybdenum disulfide; N–GQDs: Nitrogen-doped GQDs; p-ABA: p-amino benzoic acid; PDA: Polydopamine; ss-DNA: Single-stranded DNA.

**Table 3:** Various other ECL-sensors for biomedical applications (2015 onwards).

Electrode	Nanomaterials	Receptor Type	Receptor	Target Analyte	Specimen	Linear Concentration Range	Detection Limit	Reference
GCE	HM–S–GQDs	Nucleic acid	Hemin/G-quadruplex DNAzyme	p53-gene	Buffer	100 fM–100 nM	66 nM	[214]
GCE	GQDs	Oligosaccharide	β-CD	L-Tyr; D-Tyr	Human serum	6–100 µM 0.1–1 mM	6.07 × 10^−9^ M 1.03 × 10^−7^ M	[215]
GCE	N–GQDs@SiO_2_ NPs	Nucleic acid	Aptamer	OTA	Peanut sample	1 × 10^−3^–10 ng mL^−1^	0.5 × 10^−3^ ng mL^−1^	[216]
GCE	MoS_2_‒GQDs	MIP	MCPA imprinted polymer	MCPA	Lake water; Tap water; Oat samples	10 pM L^−1^–0.1 µM L^−1^	5.5 pM L^−1^	[217]
GE	GQDs	Nucleic acid	DTC-DNA probe	HCV- 1b	Buffer	5 fM–100 pM	0.45 fM	[218]
GCE	NH_2_–PTCA/Au@Fe_3_O_4_/GQDs	Nucleic acid	DNA	miRNA	Buffer; Hela, HK-2, LO2, 22Rvl cell lines	2.5 fM–50 pM	0.83 fM	[219]
GCE	AuNPs/HM-S‒GQDs	Antibody	Anti-CEA	CEA	Buffer	0.02–80 × 10^−9^ g mL^−1^	0.01 × 10^−9^ g mL^−1^	[220]
Pt.	B–GQDs@AuNPs	Nucleic acid	DNA	miRNA-20 a	Human serum	0.1–1 × 10^4^ pM	0.1 pM	[221]
GCE	Ag_core_@Au_shell_ NPs/GQDs	Nucleic acid	DNA	lnc-RNA	Human serum	1 fM–5 nM	0.3 fM	[222]

Abbreviations: 22Rvl: Prostate carcinoma cell line; β-CD: β-Cyclodextrin; AuNPs: Gold nanoparticles; B‒GQDs: Boron-doped GQDs; CEA: Carcinoembryonic antigen; DNA: Deoxyribonucleic acid; DPV: Differential pulse voltammetry; DTC: Dithiocarbamate; Fe3O4: Ferrous ferric oxide; GCE: Glassy carbon electrode; GE: Glass electrode; GQDs: Graphene quantum dots; HCV-1b: Hepatitis-C virus- 1b; Hela: Cervical cancer cell line; Hg^2+^: Mercury (II) ion; HK-2: Human renal cubular epithelial cell line; HM-S‒GQDs: Hydrazine modified single layer GQDs; MCPA: 2-methyl 4-chlorophenoxyacetic acid; MIP: Molecularly imprinted polymer; miRNA: microRNA; MoS2‒GQDs: Molybdenum sulfide GQDs; N‒GQDs: Nitrogen-doped GQDs; NH2–PTCA: aminated–3,4,9,10–Perylenetetracarboxylic acid; lnc-RNA: long non-coding RNA LO2: Normal hepatocyte cell line; OTA: Ochratoxin-A; p53: Tumor protein-53; Pt.: Platinum; SiO2 NPs: Silica nanoparticles; Tyr: Tyrosine.

**Table 4:** Various GQD-based voltammetric sensors reported (2017 onwards) for biomedical applications.

Electrode	Nanomaterials	Receptor Type	Receptor	Target Analyte	Detection Technique(s)	Specimen	Linear Range	Detection Limit	Reference
GCE	GQDs	Enzyme	Laccase	EP	CV	Pharmaceuticals	1–120 µM	83 nM	[292]
GCE	N–GQDs	Oligosaccharide	β-CD	Cholesterol	DPV	Buffer, human serum	0.5–100 nM	80 nM	[293]
GCE	GQDs	Nucleic acid	DNA probe	HBV	CV; DPV	Human serum	10 –500 nM	1 nM	[294]
GCE	C_3_N_4_NTs@GQDs	MIP	CHL-imprinted polymer	CHL	SWV	Waste water	0.01–1 nM	2 pM	[295]
SPGE	PAMAM/GQDs/AuNPs@MWCNTs	Antibody	Anti-tTG	tTG	CV; DPV	Buffer; human serum	20 fg mL^−1^–2 µg mL^−1^	20 fg mL^−1^	[76]
GCE	Ru_(core)_@Au_(shell)_	MIP	PEA-imprinted polymer	PEA	CV; DPV	Urine	1 pM–1 nM	0.2 pM	[296]
GCE	AuPd NWs/D‒GQDs–GMA	Nucleic acid	DNA probe	Ara h1	DPV	Peanut milk	1 × 10^−22^–1 × 10^−17^ M	4 × 10^−23^ M	[297]
GCE	GQDs/PdNPs	MIP	CIT-imprinted polymer	CIT	CV; DPV	Chicken egg	1–5 nM	0.2 nM	[298]
GCE	GQDs	Enzyme	Uric oxidase	UA	CV	Human serum	1–800 µM	0.3 µM	[299]
SPCE	GQDs implanted with N-ABA	MIP	Ifsosfamide-imprinted polymer	IFO	DPV	Aq. Solution; Blood plasma; Urine; Pharmaceuticals	0.25–121 ng mL^−1^ 0.31–116 ng mL^−1^ 0.28–110 ng mL^−1^ 0.32–109 ng mL^−1^	0.08 ng mL^−1^ 0.11 ng mL^−1^ 0.10 ng mL^−1^ 0.10 ng mL^−1^	[300]
GCE	NH_2_–GQDs/PBP_(electroactive label)_	Nucleic acid	ss-ds-DNA probes	micro RNA-25	DPV	Spiked human serum	0.3 nM–1 µM	95 pM	[301]
GCE	GQDs	Enzyme	Glucose oxidase	Glucose	CV; DPV	Buffer	10 µM–3 mM	1.35 µM	[16]
GCE	GQDS/hNiNs	MIP	BPS-imprinted polymer	BPS	DPV	Mineral water; extracted plastic solution	0.1–50 µM	0.03 µM	[302]

Abbreviations: β-CD: β-Cyclodextrin; Ara h1: Peanut allergen; Aq.: Aqueous; AuNPs: Gold nanoparticles; BPS: Bisphenol S; C3N4NTs: Carbon nitride nanotubes; CHL: Chlorpyrifos; CIT: Citrinin; D‒GQDs: Dodecylamine‒GQDs; DNA: Deoxyribonucleic acid; EP: Epinephrine; GMA: graphene micro-aerogel; HBV: Hepatitis-B virus; hNiNs: Hollow nickel nanospheres; IFO: Ifsosfamide; MIP: Molecularly imprinted polymer; MWCNTs: Multi-walled carbon nanotubes; N-ABA: N-Acryloyl-4-aminobenzamide; N–GQDs: Nitrogen-doped GQDs; NWs: Nano-waxberries; PAMAM: Polyamidoamine; PBP: p-Biphenol Pd: Palladium; PEA: Phenyl-ethanolamine; Ru: Ruthenium; ss-ds-DNA: Single- and double-stranded DNA; SWV: Square wave voltammetry; tTG: tissue transglutaminase; UA: Uric acid.
